# ChLae1 and ChVel1 Regulate T-toxin Production, Virulence, Oxidative Stress Response, and Development of the Maize Pathogen *Cochliobolus heterostrophus*

**DOI:** 10.1371/journal.ppat.1002542

**Published:** 2012-02-23

**Authors:** Dongliang Wu, Shinichi Oide, Ning Zhang, May Yee Choi, B. Gillian Turgeon

**Affiliations:** 1 Dept. of Plant Pathology & Plant-Microbe Biology, Cornell University, Ithaca, New York, United States of America; 2 Molecular Microbiology and Biotechnology group, Research Institute of Innovative Technology for the Earth, Kyoto, Japan; 3 Dept. of Plant Biology & Pathology, Rutgers University, New Brunswick, New Jersey, United States of America; Virginia Polytechnic Institute and State University, United States of America

## Abstract

LaeA and VeA coordinate secondary metabolism and differentiation in response to light signals in *Aspergillus* spp. Their orthologs, ChLae1 and ChVel1, were identified in the maize pathogen *Cochliobolus heterostrophus*, known to produce a wealth of secondary metabolites, including the host selective toxin, T-toxin. Produced by race T, T-toxin promotes high virulence to maize carrying Texas male sterile cytoplasm (T-cms). T-toxin production is significantly increased in the dark in wild type (WT), whereas *Chvel1* and *Chlae1* mutant toxin levels are much reduced in the dark compared to WT. Correspondingly, expression of T-toxin biosynthetic genes (*Tox1*) is up-regulated in the dark in WT, while dark-induced expression is much reduced/minimal in *Chvel1* and *Chlae1* mutants. Toxin production and *Tox1* gene expression are increased in *ChVEL1* overexpression (OE) strains grown in the dark and in *ChLAE1* strains grown in either light or dark, compared to WT. These observations establish ChLae1 and ChVel1 as the first factors known to regulate host selective toxin production. Virulence of *Chlae1* and *Chvel1* mutants and OE strains is altered on both T-cms and normal cytoplasm maize, indicating that both T-toxin mediated super virulence and basic pathogenic ability are affected. Deletion of *ChLAE1* or *ChVEL1* reduces tolerance to H_2_O_2_. Expression of *CAT3*, one of the three catalase genes, is reduced in the *Chvel1* mutant. *Chlae1* and *Chvel1* mutants also show decreased aerial hyphal growth, increased asexual sporulation and female sterility. *ChLAE1* OE strains are female sterile, while *ChVEL1* OE strains are more fertile than WT. ChLae1 and ChVel1 repress expression of 1,8-dihydroxynaphthalene (DHN) melanin biosynthesis genes, and, accordingly, melanization is enhanced in *Chlae1* and *Chvel1* mutants, and reduced in OE strains. Thus, ChLae1 and ChVel1 positively regulate T-toxin biosynthesis, pathogenicity and super virulence, oxidative stress responses, sexual development, and aerial hyphal growth, and negatively control melanin biosynthesis and asexual differentiation.

## Author Summary

Filamentous fungi produce chemically diverse metabolites that broker positive and negative interactions with other organisms, manage host pathogenicity/virulence, nutritional and environmental stresses, and differentiation of the fungus. The maize pathogen *Cochliobolus heterostrophus* is notorious as the causal agent of the most economically devastating epidemic to date, in 1970. Disease severity was associated with appearance of a new race, producing T-toxin, a host selective toxin promoting high virulence to Texas male sterile cytoplasm maize, widely planted at the time. LaeA and VeA are central regulators of secondary metabolism in *Aspergillus*, coordinating metabolite production and differentiation in response to light. Given the significance of effector-type host selective toxins in pathogenic interactions, we characterized ChLae1 and ChVel1 and found that deletion and overexpression affect T-toxin production *in planta* and *in vitro*. Both chlorosis due to T-toxin and necrotic lesion formation are altered, establishing these as the first factors known to regulate both super virulence conferred by T-toxin, and basic pathogenicity, due to unknown factors. The mutants are also altered in oxidative stress responses, key to success in the infection court, asexual and sexual development, essential for fungal dissemination in the field, aerial hyphal growth, and pigment biosynthesis, essential for survival in the field.

## Introduction

In filamentous fungi, development and secondary metabolism are intimately coordinated [1], [2]. Two proteins, VeA and LaeA have been demonstrated to be centrally involved in orchestration of these fundamental processes for a handful of fungi such as the eurotiomycetes *Aspergillus* spp., and *Penicillium* spp. [3]–[5], and the sordariomycetes, *Acremonium chrysogenum* [6] and *Fusarium* spp. [7], [8]. VeA was first identified as a positive regulator of sexual sporulation and negative regulator of asexual development in *Aspergillus nidulans* [9]. Later, it was found to play a role also in positive regulation of sterigmatocystin (ST) and penicillin biosynthesis [3]. The VeA orthologs have been reported to control biosynthesis of various secondary metabolites such as aflatoxin, cyclopiazonic acid, and aflatrem by *Aspergillus flavus* [10] gibberellin (GA), fumonisin, fusarin C, and bikaverin by *Fusarium fujikuroi* [8], fumonisin and fusarin by *Fusarium verticillioides* [7] and trichothecenes by *Fusarium graminearum* [11]. LaeA was first characterized as a global regulator of secondary metabolism in *A. nidulans*; like *veA* mutants, loss of functional LaeA leads to reduced production of ST, penicillin, and lovastatin [12]. The LaeA orthologs have been identified and characterized in several filamentous ascomycetes. Microarray analysis of *Aspergillus fumigatus* wild type (WT) and *laeA* mutants demonstrated that LaeA controls 13 of 22 secondary metabolite gene clusters [13]; similar analyses with *A. flavus* revealed that at least 20 of 55 clusters were reduced in a *laeA* deletion mutant [14]. Recently, LaeA was reported also to play a role in asexual and sexual development of *A. nidulans* [15], and asexual development in *Penicillium chrysogenum* [5] and *F. fujikuroi* [8].

In terms of mechanism, in *A. nidulans*, VeA and LaeA physically interact with each other forming the so-called velvet complex with a third protein, VelB, to regulate secondary metabolism and fungal differentiation in response to light signals [4]. VeA also interacts with the red light sensing protein FphA that is associated with the blue light receptors LreA and LreB. [16]. Physical interaction of the VeA/LaeA orthologs, Vel1/Lae1, in the nucleus under dark conditions has been reported also for *F. fujikuroi* [8]. LaeA is a putative methyltransferase, while VeA is a presumed scaffold protein that collaborates with other proteins (FphA, VelB, etc) to transduce extracellular cues such as light signals to the nucleus [16], [17].

In *A. nidulans*, reproductive development and secondary metabolite biosynthesis are controlled in a light-dependent manner [15], [16], [18]. Sexual development and secondary metabolism are induced in dark and repressed in light, while asexual sporulation (i.e. conidiation) is regulated in a contrasting way in response to a light/dark signal. Protein level and subcellular localization of VeA are considered to be the key for induction of sexual development and secondary metabolism in the dark. In the light, VeA protein levels are reduced compared to levels in the dark, and the protein is found mostly in the cytoplasm. VeA is transferred to the nucleus in the dark where it forms the complex with VelB and LaeA to turn on genes involved in sexual development and secondary metabolism [4], [15], [19].

The phenotype of *F. fujikuroi vel1* and *lae1* deletion mutants generally mirror those of *Aspergillus* spp. but with some twists; the Vel1 protein, for example, can act as both a positive (GAs, fumonisins and fusarin C) and a negative (bikaverin) regulator of secondary metabolism. With respect to plant pathogenicity, deletion of either *F. fujikuroi Vel1* or *Lae1* causes altered symptoms on plants, (most likely due to GA reduction), suggesting they are virulence determinants [8]. Earlier, Duran et al. demonstrated that *A. flavus veA* mutants are less able to colonize corn seeds [10]. Recently, this was also observed for colonization of maize seedlings by *F. verticillioides* [20]. In *F. graminearum*, a *Fgve1* mutant showed reduced virulence to wheat spikelets [11]. In terms of impact on animal pathogenicity, *A. fumigatus laeA* mutant spores are more easily taken up by phagocytosis, apparently because they produce less RodA hydrophobin protein [21], but not because secondary metabolite production is affected.

*Cochliobolus heterostrophus*, a necrotrophic dothideomycete pathogen of maize, produces a large and diverse array of secondary metabolites biosynthesized by 25 polyketide synthases (PKS) and 14 nonribosomal peptide synthetases (NRPS). Heterothallic *C. heterostrophus* and related species have been used as models for understanding reproductive evolution and development [22], [23]. To date the importance of VeA and LaeA orthologous proteins in regulation/coordination of secondary metabolism, virulence associated with host selective toxin (HST)/effector biosynthesis, and development, has not been examined in *Cochliobolus* or in closely related dothideomycete taxa such as *Pyrenophora* and *Alternaria* spp., fungi well-known for their impact on our staple crops, especially cereals. These fungi are notorious for production of HSTs, the first well-characterized fungal virulence effectors, recognized because their producing fungi contributed to decimation of US agricultural crops. We were particularly interested in examining whether or not LaeA and VeA homologs regulate the HST T-toxin, produced by *C. heterostrophus* race T as it is a well-documented, critical metabolite in its interaction with maize, but has a complex, as yet unknown evolutionary history. Race T had not been recognized until it caused an epidemic of Southern Corn Leaf Blight (SCLB) in 1970 [24]. On maize cultivars with Texas male-sterile cytoplasm (T-cms), race T develops devastating symptoms, distinguishing itself from mildly pathogenic race O, prevalent before the 1970's outbreak of SCLB. The highly aggressive nature of race T is solely attributable to its ability to produce T-toxin, a family of linear polyketides, which requires two PKSs (PKS1 and PKS2) [25] and at least seven other proteins for its biosynthesis [26], [27]. The T-toxin biosynthetic genes (encoded at the *Tox1* locus), are present only in race T and not in race O, and are not tightly clustered in contrast to most described fungal secondary metabolite genes, especially those in the *Aspergillus* and *Fusarium* species described above [28]–[30]. The *Tox1* genes reside at two different loci inseparably associated with the breakpoints of a reciprocal translocation on two different chromosomes. At one of these loci, three small contigs carrying T-toxin genes have been identified, however, although they map genetically to the same locus, these contigs cannot be connected together by sequencing, likely due to the highly repetitive A+T-rich nature of their flanking DNA. Of the nine *Tox1* genes, only two appear to be related phylogenetically [27]. To date, no regulatory protein, specific or otherwise has been identified associated with T-toxin biosynthesis. Our first goal in this study was to ask if VeA and LaeA orthologous proteins are involved in regulation of this genetically and phylogenetically complex HST secondary metabolite.

A second, central virulence-associated secondary metabolite described for *C. heterostrophus* is the extracellular siderophore biosynthesized by the NRPS, NPS6. Deletion of *NPS6* from necrotrophic *C. heterostrophus*, *Cochliobolus miyabeanus*, *Gibberella zeae/Fusarium graminearum*, and *Alternaria brassiciciola*, causes reduced virulence to the corresponding hosts (maize, rice, wheat and Arabidopsis), and hypersensitivity to H_2_O_2_ and to low iron [31], [32]. Iron is an essential nutrient and it is intrinsically linked to reactive oxygen species (ROS) which serve as antimicrobials and signals in plant-microbe interactions. Iron has the potential to catalyze the Fenton/Haber Weiss reactions [33] that generate highly cytotoxic ROS, hence, mechanisms that sequester iron in cells are critical to the survival of all organisms. Here we have examined whether or not NPS6, a metabolite produced by a gene associated with so-called secondary metabolism but critical for cellular management of the essential nutrient iron, is controlled by *A. nidulans* LaeA and VeA orthologous proteins in *C. heterostrophus*.

A third metabolite that we examined is melanin, which, like T-toxin, is the product of a PKS. Melanin biosynthesis is essential for survival of *C. heterostrophus* in the field but not under laboratory conditions [34]. In many fungi, pigment, often melanin, is directly associated with developmental structures as a cell wall component of hyphae and asexual and sexual spores [35]–[39]; loss of pigment is associated with increased susceptibility to ROS [40] in *A. fumigatus* and *A. nidulans*. A recent report documents that the ortholog of VeA in *Mycosphaerella graminicola*, a hemibiotrophic dothideomycete pathogen of wheat, positively regulates melanin biosynthesis and aerial mycelial growth [41].

In this study, we identified, deleted, and overexpressed the *LaeA* and *VeA* orthologs (designated *ChLAE1* and *ChVEL1*) in *C. heterostrophus* and showed that both play roles in T-toxin production and virulence in response to light conditions, despite the complex genomic structure and uncertain evolutionary history of this HST/effector. In addition, ChLae1 and ChVel1 positively regulate oxidative stress tolerance, sexual development, and aerial hyphal growth, and negatively control asexual development and expression of melanin biosynthesis genes. Expression of the extracellular siderophore- associated *NPS6* gene, key to survival in the infection court, was unaffected in *Chlae1* or *Chvel1* mutants.

## Results

### Identification of the LaeA and VeA orthologs in *C. heterostrophus*

Several candidate homologs were identified in the C5 genome database when queried with *A. nidulans* LaeA. The candidate with the highest score (153), lowest E value (2×e^−87^) and with the closest phylogenetic placement to *A. nidulans* LaeA was chosen for functional analyses (named *ChLAE1*, accession number JF826792). Phylogenetic analysis indicated that the ChLae1 protein falls in a well-supported group of dothideomycete LaeA homologs (Figure S1A). This group is sister to the eurotiomycete group that includes *A. nidulans* LaeA and does not group with additional *C. heterostrophus* LaeA like proteins which are also found in *A. nidulans* (e.g., AnLlmF) and other filamentous fungi [42]. Alignment of ChLae1 with *A. nidulans* LaeA (Figure S1B) showed 51% identity and 67% positives (NCBI BlastP Align). Alignment of LaeA homologs from *A. nidulans*, *A. fumigatus*, and *C. heterostrophus* using ClustalW identified a highly conserved SAM-dependent methyltransferase domain, characteristic of LaeA proteins (Figure S1B).

A single candidate VeA ortholog (*ChVEL1*, accession number JF826791) was identified when the *C. heterostrophus* genome was queried with *A. nidulans* VeA (score of 92 and E value of 9×e^−62^). Phylogenetic analysis indicated that the ChVel1 protein falls in a well-supported group of dothideomycete VeA homologs (Figure S2A), which is sister to the eurotiomycete group including *A. nidulans* VeA. ChVel1 has 53% identity and 66% positives with AnVeA (Figure S2B). A putative pat7 nuclear localization signal (NLS) and bipartite NLS found in the *Aspergillus* spp. proteins [19] were not found in ChVel1 using the Wolf PSORT (http://www.wolfpsort.org; [43]) subcellular localization prediction program. However, the cNLS Mapper program (http://nls-mapper.iab.keio.ac.jp/, [44]) identified a potential α importin-dependent monopartite NLS at the C-terminus of ChVel1. A PEST (proline, glutamate, serine and threonine-rich region) motif was predicted also at the C-terminus of ChVel1.

### ChLae1 and ChVel1 regulate T-toxin biosynthesis

**Figure 1:**
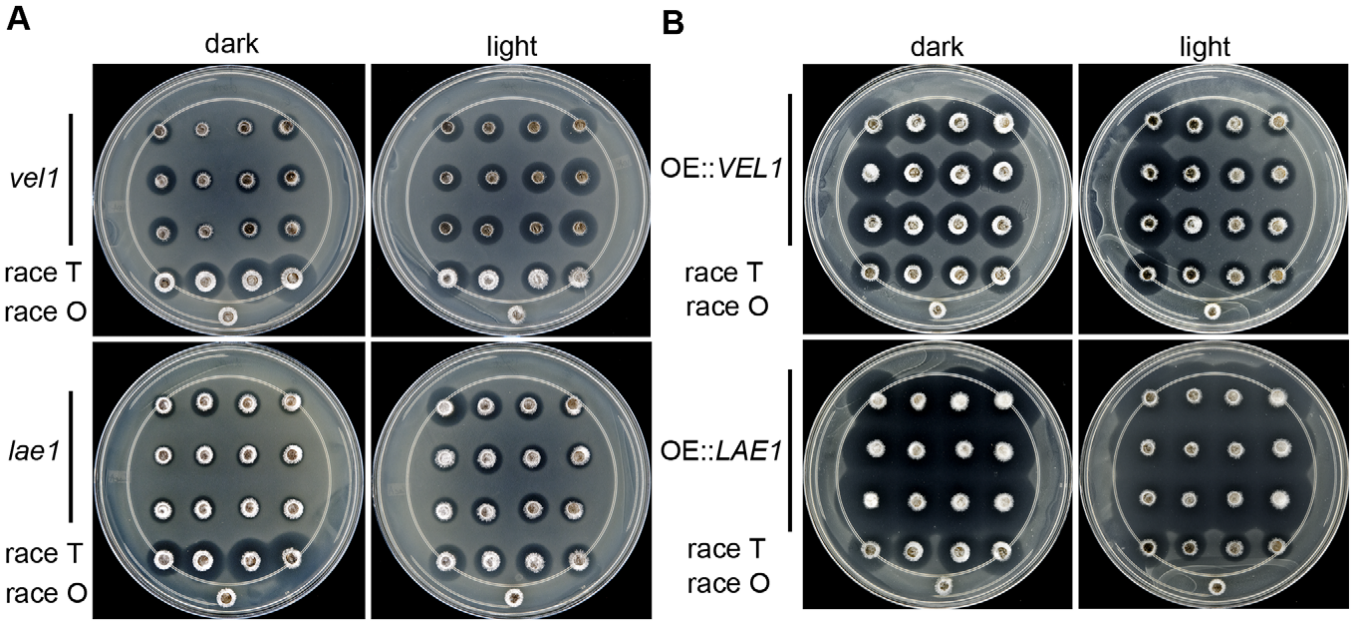
*Chlae1* and *Chvel1* mutants make less, or less active, T-toxin and *ChVEL1* and *ChLAE1* overexpression strains make more T-toxin in constant dark than wild-type strains. **A.** Microbial assay plates for T-toxin production. Plates were spread with *E. coli* cells carrying the *URF13* gene that confers sensitivity to T-toxin. Plugs of each fungal strain grown for eight days at 19°C on CMX medium under constant dark and constant light conditions, were placed on the plates, mycelium side up and incubated overnight. Clear area (halo) indicates T-toxin production and killing of *E. coli* cells. On each plate, the bottom single plug is a race O, T-toxin^−^ control (strain C5, no halo). Second row from bottom is race T, T-toxin^+^ control (strain C4, halos). Top three rows are three replicates of the mutant strain indicated. From left to right in each row are plugs of mycelium taken from the center to the edge of the colony. Note that the halo sizes around WT strain C4 are comparable in light, however, in the dark they are larger when plugs were taken from the outer (younger) edges of the colony. Both mutants form smaller halos compared to WT grown in the dark, howeve,r halos around *Chvel1* mutants are almost as big as those of WT grown in the light. **B.** *ChVEL1* and *ChLAE1* overexpression strains grown on CMX medium supplemented with polygalacturonic acid (PGA) as described in A. Plates were set up as in A, except that overexpression strains were assayed instead of mutants. The *LAE1* overexpressing strain produced more T-toxin than WT under both light and dark conditions while the *VEL1* overexpressing strain produced more T-toxin than WT in the dark, but not in the light.

**Figure 2:**
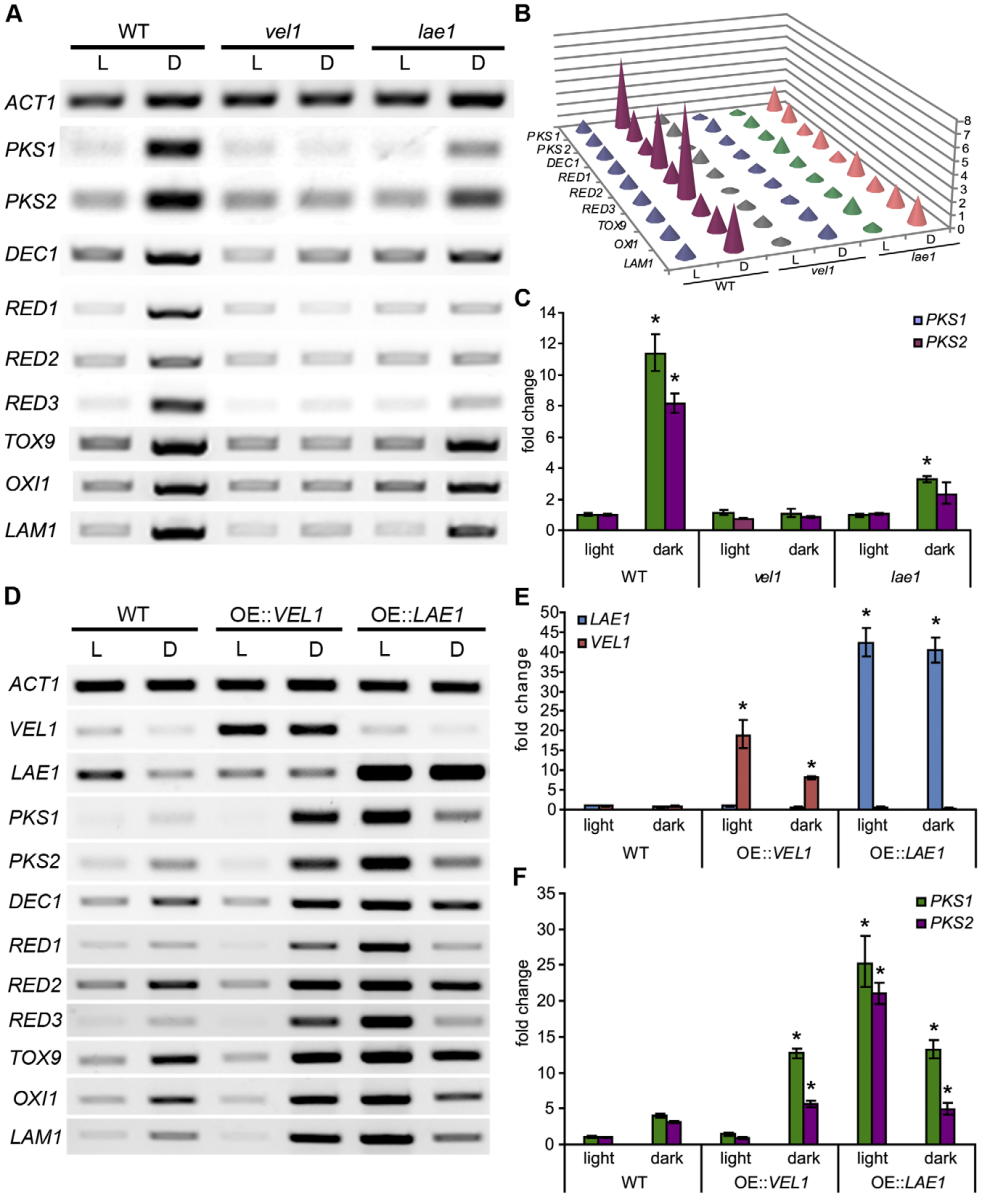
ChLae1 and ChVel1 positively regulate T-toxin biosynthesis genes. **A.** RT-PCR analysis of the genes known to be involved in T-toxin production in WT and mutant strains. Expression of *ACT1* indicates relative RNA quantity in each sample. All *Tox1* genes are up-regulated in the dark, relative to in the light in WT. Evidence of light regulation is erased in *Chvel1* mutants. Most genes are weakly up-regulated in *Chlae1* mutants, except for *RED1*, *RED2* and *RED3*. **B.** Quantification of band intensity. Band intensity in Figure 2A was quantified by Image J. The band intensity ratio of each *Tox1* gene and that of the corresponding control *ACT1* gene was calculated and normalized to that of WT strain C4 in light. Note that band intensity of all genes is elevated in the dark in WT, while band intensity of all genes is minimal in the *Chvel1* mutant grown in both the light and dark, and minimally elevated in the *Chlae1* mutant grown in the dark. **C**. qPCR of *PKS1* and *PKS2*. Error bars represent range of fold change calculated according to standard deviation of ΔΔCt. Asterisks indicate p-value <0.001 in T-test analysis in which all the strains grown in the dark were compared to their corresponding strain grown in constant light. **D.** RT-PCR analysis of *Tox1* genes in overexpression strains. Overexpression of *ChLAE1* results in drastic up-regulation of all genes in the light and a moderate increase in the dark compared to WT. In contrast, *ChVEL1* overexpression caused up-regulation of *Tox1* gene expression in the dark but not the light. **E.** qPCR of *ChLAE1* and *ChVEL1*. cDNA samples are the same as A. Error bars represent range of fold change calculated according to standard deviation of ΔΔCt. Asterisks indicate p-value <0.01 in T-test analysis in which both overexpression strains were compared to WT grown in the same condition. **F**. qPCR of *PKS1* and *PKS2*. Same as B. Asterisks indicate p-value <0.05 in T-test analysis in which both overexpression strains were compared to WT grown in the same condition.

The effect of *ChLAE1* and *ChVEL1* deletions on T-toxin production in constant light and constant dark conditions was examined using the microbial assay [45]. T-toxin was produced by WT race T strain C4 under both light conditions tested, but much more so in the dark (Figure 1) and increasingly as the sample was taken from the center to the edge of 8 day old colonies (Figure 1A, lower left). No T-toxin was produced by control WT race O strain C5. In dark conditions, smaller halos were observed for *Chlae1* and *Chvel1* mutants compared to WT race T. In light conditions, this was still the case for *Chlae1* mutants, while *Chvel1* mutant plugs had bigger halos than in the dark and those taken from the edge of the colony were slightly larger than those of WT in the light (Figure 1A, top right). Overexpression of *ChVEL1* caused a modest increase in T-toxin production in the dark (Figure 1B, top row), but not in the light when compared to WT strains. Overexpression of *ChLAE1* lead to profuse over-production of T-toxin in both the dark and light compared to WT (Figure 1B, bottom row). These data suggest that both ChLae1 and ChVel1 positively regulate T-toxin production under dark conditions, however the observation that some toxin is produced by both mutants suggests that there must be an additional T-toxin biosynthesis regulatory mechanism(s) that is independent of ChLae1 and ChVel1. Copious production of toxin in the light by *LAE1* overexpression strains suggests that regulatory mechanisms associated with light signals were disturbed. To further confirm that ChLae1 and ChVel1 act as positive regulators of T-toxin production, expression of all nine known *Tox1* genes (*PKS1*, *PKS2*, *DEC1*, *RED1*, *RED2*, *RED3*, *TOX9*, *OXI1*, *LAM1*) [25]–[27] was examined for WT race T strain C4, *Chlae1*, and *Chvel1* strains grown in constant light and constant dark at 19°C, the same conditions as culture plates used for the T-toxin assay (Figure 1). All *Tox1* genes were up-regulated in the dark in WT, whereas dark-induced expression was not evident or reduced in *Chvel1* and *Chlae1* mutants, respectively (Figure 2A). Quantification of the RT PCR bands in Figure 2A showed that for *Chvel1* mutants, most genes were not up-regulated, or were minimally up-regulated, in the dark. For *Chlae1* mutants, expression levels of the nine genes were slightly more elevated than in *Chvel1* mutants in the dark but in all cases less so than for WT in the dark (Figure 2B). We also carried out quantitative PCR (qPCR) analyses on the core *PKS1* and *PKS2* genes required for T-toxin production (Figure 2C). The two *PKS*s were minimally expressed in light and induced in dark in WT strain C4 (Figure 2C) and in *laeA*[*LAE1*] and *vel1*[*VEL1*]complemented strains (not shown). In contrast, expression of these genes in *Chvel1* mutants was like that of WT in the light, regardless of the light conditions. Expression of these genes in *Chlae1* mutants was slightly up in the dark compared to expression in the light, but significantly less than that of WT in the dark (Figure 2C). These results are consistent with the T-toxin microbial assay, in which T-toxin production was significantly decreased in *Chlae1* and *Chvel1* mutants grown under dark conditions, and demonstrate that ChLae1 and ChVel1 are required for positive regulation of T-toxin genes in the dark.

Expression levels of *ChLAE1* and *ChVEL1* genes were clearly upshifted (Figure 2E) in *ChLAE1* and *ChVEL1* overexpression strains. Overexpression of *ChVEL1* caused a modest increase in T-toxin gene expression in the dark, but not in the light when compared to WT strains (Figure 2D), consistent with toxin production observations (Figure 1B). In strains overexpressing *ChLAE1*, all or most genes involved in T-toxin production were up-regulated in the dark compared to WT (Figure 2D). In contrast to the *LAE1* overexpression strain grown in the dark, and to WT and *VEL1* overexpression strains in the light, expression of all toxin genes was strongly upregulated in the light in *LAE1* overexpression strains (Figure 2D), in keeping with profuse T-toxin production by these strains (Figure 1B, bottom right). These data demonstrate that *ChLAE1*and *ChVEL1* regulate T-toxin production through transcriptional control of *Tox1* genes.

### ChLae1 and ChVel1 are important for virulence to maize carrying normal, as well as T-cytoplasm

**Figure 3:**
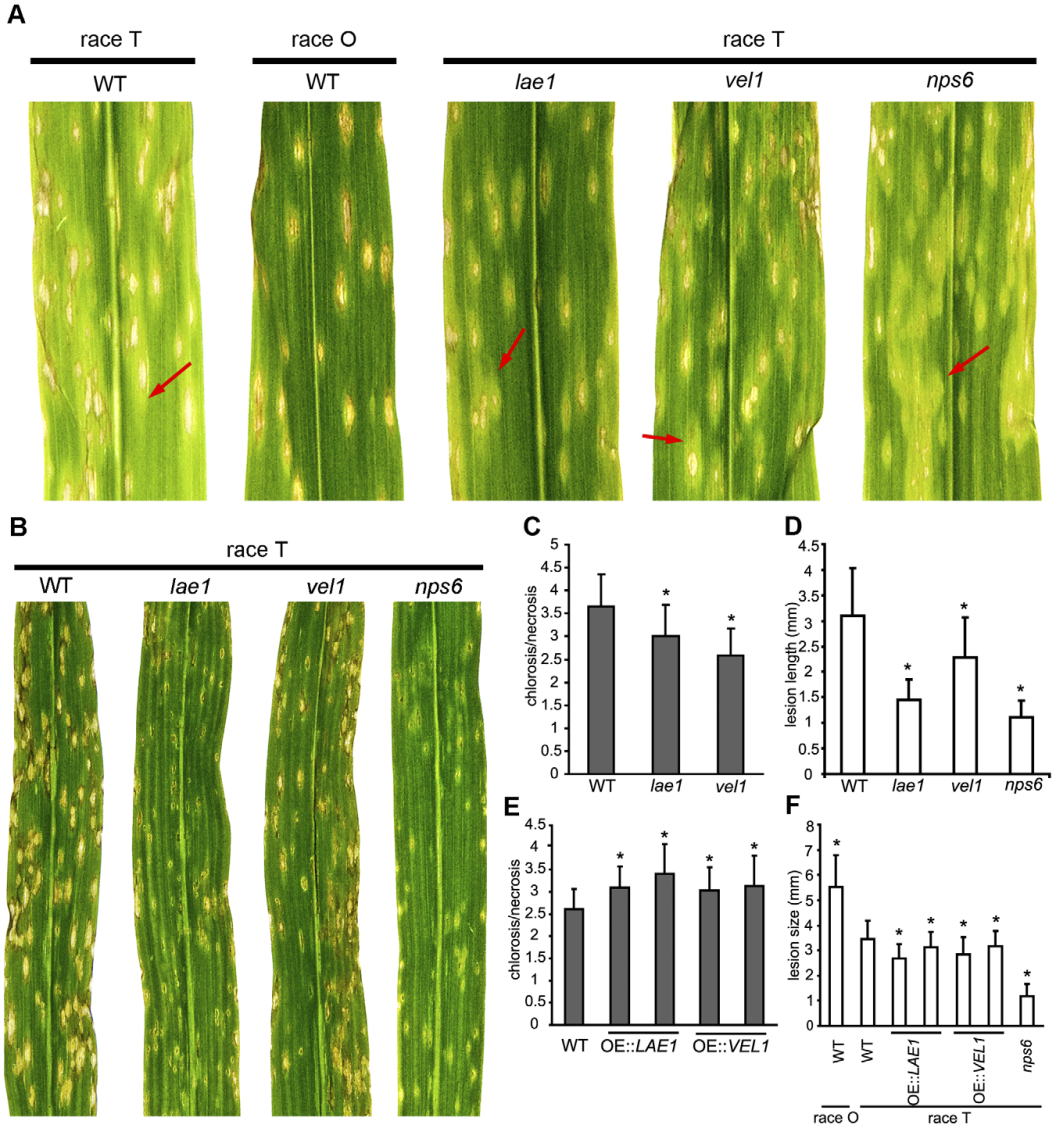
*Chlae1* and *Chvel1* mutants show reduced virulence to maize with T- and N-cytoplasms. **A.** Virulence on T-toxin sensitive T-cytoplasm corn. Leaves spray-inoculated with WT race T strain C4, the *nps6* mutant lacking extracellular siderophores (strain Chnps6-3), *Chvel1* mutant (strain ChW7), *Chlae1* mutant (strain ChW5), and T-toxin^−^, race O control strain C5. WT C4 shows chlorotic halos around beige lesion cores and chlorotic streaking (red arrow) due to T-toxin production. Control race O strain C5 makes defined beige-light brown lesions and no chlorotic streaking. The *nps6* mutant makes tiny beige lesions, but lots of chlorosis (red arrow) due to T-toxin production. *Chvel1* and *Chlae1* mutants show reduced lesion size compared to WT and the amount of chlorosis is less than that of WT or the *nps6* strain (red arrows). **B.** Virulence on T-toxin insensitive N-cytoplasm corn leaves. Leaves spray-inoculated with the same set of strains as in **A**, except for strain C5. On N-cytoplasm, *Chvel1* and especially *Chlae1*, mutants show reduced lesion size compared to WT but in both cases lesions are less reduced in size than those of the *nps6* strain. **C.** Quantification of disease on T-cytoplasm leaves shown in **A**. The length of the chlorosis and necrosis symptom associated with individual lesions was measured and the average chlorosis/necrosis ratio plotted. Error bars are standard deviation. Asterisks represent p-value <0.05 in T-test analysis when each mutant was compared with WT C4. **D.** Quantification of lesion sizes of N-cytoplasm leaves shown in **B**. The length of the necrosis lesion symptom associated with individual lesions was measured and the average plotted. Error bars are standard deviation. Asterisks represent p-value <0.05 in T-test analysis when each mutant was compared with WT C4. **E.** Quantification of disease on T-cms corn leaves caused by *ChVEL1* and *ChLAE1* overexpression strains, using methods described in **C** above. **F.** Quantification of lesion sizes on N-cytoplasm corn leaves caused by *ChVEL1* and *ChLAE1* overexpression strains, using methods described in **D** above.

Maize cultivars carrying T-cms are inherently sensitive to T-toxin, while those with normal (N) cytoplasm are insensitive. Race T is able to infect plants with both types of cytoplasm but shows highly enhanced virulence to T-cms plants. On these, WT race T strain C4 shows extensive chlorosis, streaking, and bleaching around beige lesion cores and the overall color of the leaf is light green-yellow (Figure 3A, left). Isogenic control race O strain C5 makes lesions that are more brown than beige and does not exhibit chlorosis, thus the overall color of the leaf is dark green (Figure 3A, second from left). To measure disease phenotype, we used the ratio of the length of the chlorosis symptom caused by T-toxin, over the length of necrotic lesion symptom for individual lesions. Lesion sizes were reduced and the amounts of observable chlorosis were reduced upon inoculation with *Chlae1* and *Chvel1* mutants, and this was reflected in the chlorosis/necrosis index (Figure 3C). These results are in correspondence with results of the microbial and expression analyses of T-toxin production. Although *lae1* and *vel1* mutant lesions are smaller than WT, they are not as small as those of the *nps6*, extracellular siderophore mutant which still exhibited extensive chlorosis (Figure 3A, right). These observations indicate that ChLae1 and ChVel1 positively regulate T-toxin production *in planta* as well as *in vitro*.

Lesion sizes on N-cytoplasm corn leaves inoculated with *Chlae1* or *Chvel1* mutants were significantly reduced compared to WT (Figures 3B, D). In terms of core lesion size, WT>*vel1>lae1*>*nps6*, while in terms of chlorosis due to T-toxin, WT = *nps6*>*vel1>lae1*. These data suggest that ChLae1 and ChVel1 are important also for basic pathogenicity (i.e., not having to do with T-toxin production) in *C. heterostrophus* and that an unidentified secondary metabolite is involved.

Strains overexpressing *LAE1* (Figure S3) and *VEL1* (not shown), produced more T-toxin when grown on polygalacturonic acid (PGA)-containing medium (which induces the *PelA* promoter driving a second copy of each gene), than when grown on glucose-containing medium. Thus, strains carrying two copies of either *LAE1* or *VEL1* make more T-toxin, as determined by the microbial assay. Accordingly, the chlorosis/necrosis ratio was higher for the overexpression strains inoculated on T-cms maize than that of WT race T (Figure 3E).

In contrast, on N-cytoplasm, all overexpressing strains caused smaller lesions than WT race T. This fits with earlier literature [46], which demonstrated a fitness cost associated with T-toxin production. Supporting this, WT race O, which does not produce T-toxin, but is otherwise isogenic to WT race T used here, makes larger lesions (Figure 3F), than WT race T. The *nps6* mutant makes much smaller lesions, but still makes WT levels of T-toxin (Figures 3A, F).

### ChLae1 and ChVel1 promote oxidative stress tolerance

**Figure 4:**
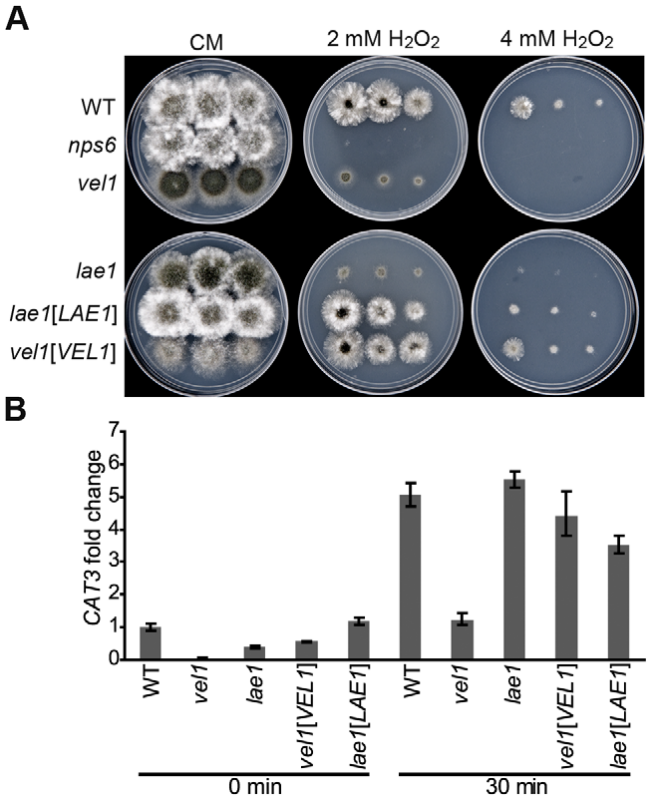
*Chlae1* and *Chvel1* mutants are hypersensitive to oxidative stress. **A**. Hypersensitivity of *Chlae1* and *Chvel1* mutants to H_2_O_2_. Serial dilutions of conidial suspensions (left to right: 4, 2 and 1 µl) prepared from WT strain C4, *nps6*, *Chvel1*, *Chlae1* mutants, and *Chvel1* (*vel1*[*VEL1*]) and *Chlae1* (*lae1*[*LAE1*]) complemented strains were placed on complete medium (CM) with and without indicated concentrations of H_2_O_2_. The *Chvel1* and *Chlae1* mutants are more sensitive to oxidative stress mediated by H_2_O_2_ than WT and the complemented strains, but not as sensitive as the extracellular siderophore *nps6* mutant [32]. **B**. qPCR analysis of *CAT3*, one of the three catalase-encoding genes in *C. heterostrophus*. *CAT3* expression was examined in the same set of strains as in **A**. Expression level relative to WT C4 at time 0 is shown. Error bars show range of fold change calculated according to standard deviation of ΔΔCt. Asterisks represent p-value <0.001 in T-test analysis in which each strain was compared with the corresponding WT C4 strain at the same time point. Deletion of *ChLAE1* does not affect expression of *CAT3*, while reduced expression was observed for *Chvel1* mutant (20 fold at time 0 and 4 fold at 30 min after H_2_O_2_ addition).

*Chlae1* and *Chvel1* mutants showed elevated sensitivity to H_2_O_2_ compared to the WT strain, but were less sensitive than the *nps6* strain which is known to be hypersensitive to oxidative stress due to lack of extracellular siderophore production [31], [32] (Figure 4A). Reintroduction of WT *ChLAE1* and *ChVEL1* genes into their corresponding mutants restored WT tolerance to oxidative stress (Figure 4A).

Expression of several *C. heterostrophus* genes associated with oxidative stress responses was analyzed using qPCR. Genes examined included *ChGSH2*, encoding glutathione synthetase, *TRX1*, *TRX2*, encoding two thioredoxins, *TRR1*, encoding thioredoxin reductase; *ChAP1*, encoding the AP1-like transcription factor [47], *CAT1*, *CAT2* and *CAT3*, encoding three catalases [48], *SKN7* and *SSK1*, encoding two different histidine kinase response regulators [2]. Of these, only *CAT3* showed a significant difference in expression level between WT and the *Chvel1* mutant (greater than three-fold change) (Figure 4B). H_2_O_2_-responsive induction, however, was observed for *GSH2*, *TRX2*, and *TRR1* in WT (5, 3.5, and 27 fold respectively), as well as in the mutants, while *ChAP1* and *SSK1* went up slightly in WT (1.4 and 2 fold, respectively) as well as in the mutants (data not shown). In the *Chvel1* mutants, *CAT3* was reduced about 20 fold at time 0, before addition of H_2_O_2_ and about 4 fold reduced after H_2_O_2_ addition compared to the level in WT. No difference in *CAT3* expression was observed between the *Chlae1* mutant and WT (Figure 4B). Overall these results suggest that ChVel1 controls oxidative stress responses, at least in part, by regulating *CAT3* expression. This mechanism needs to be explored further. Reduced virulence and increased sensitivity to H_2_O_2_ are reminiscent of characteristics of the *C. heterostrophus nps6* mutant that lacks the NRPS responsible for extracellular siderophore synthesis, although virulence and H_2_O_2_ tolerance of the *Chlae1* and *Chvel1* mutants are not as severely affected as those of the *nps6* mutant (Figures 3 and 4). To test whether *NPS6* is under the control of ChLae1 and ChVel1, we examined low iron-induced expression of *NPS6* in the *Chlae1* and *Chvel1* mutants. Under these conditions, *NPS6* was strongly induced in WT and both mutants, indicating that ChLae1 and ChVel1 do not play a role in transcriptional regulation of *NPS6*, at this level of determination (data not shown). In subsequent experiments, we determined that *LAE1* and *VEL1* expression in WT is the same in iron-replete and depleted conditions (data not shown), confirming the lack of involvement of these two regulators in managing iron.

### ChLae1 and ChVel1 control sexual development

**Figure 5:**
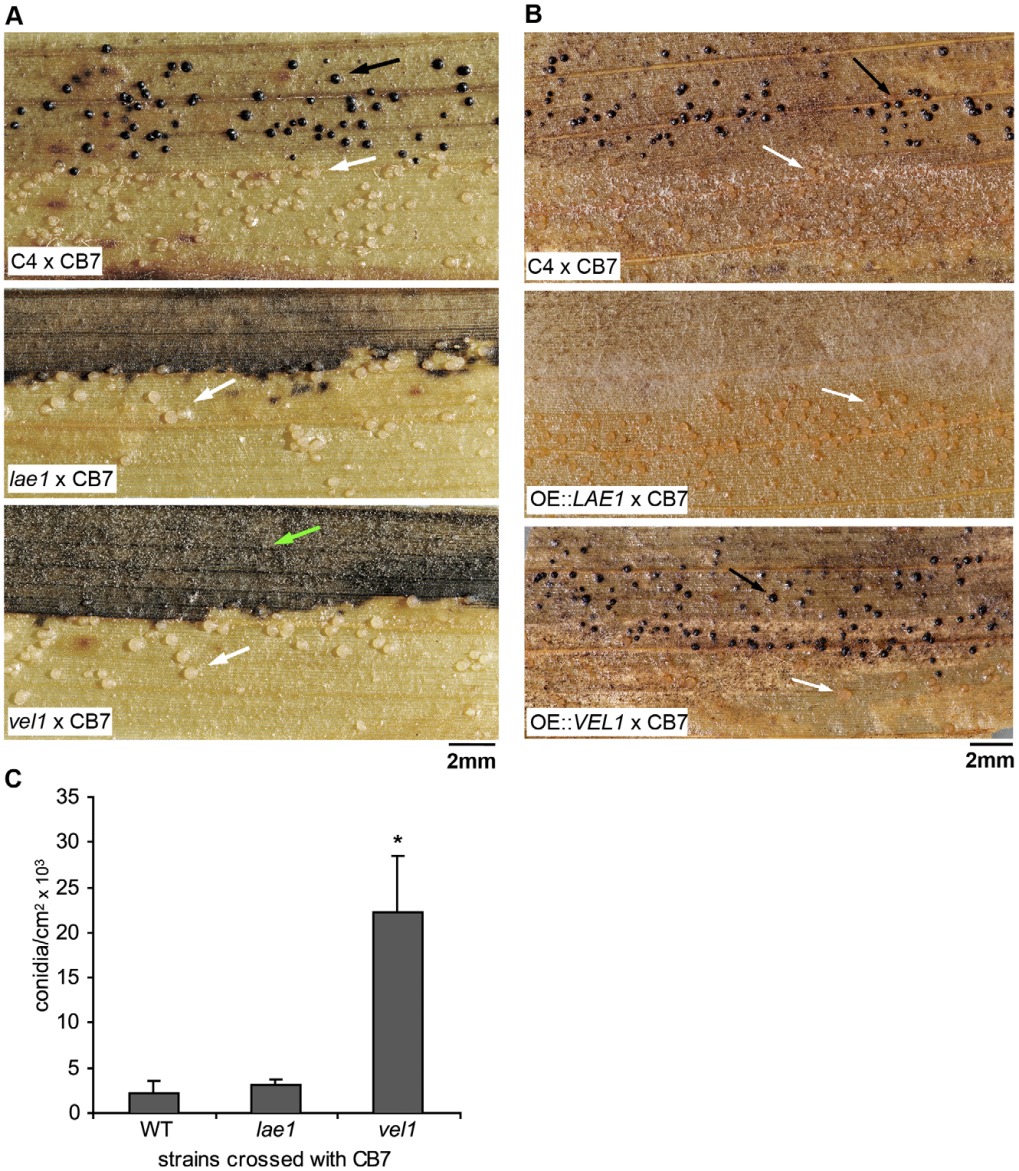
*Chlae1* and *Chvel1* mutants are compromised in reproductive development. **A.** *Chlae1* and *Chvel1* mutants are female sterile. Top: a control cross between pigmented WT strain C4 and albino WT strain CB7. Both black and white pseudothecia (arrows) are produced indicating both strains are hermaphroditic. Middle and bottom rows: crosses between albino WT strain CB7 and pigmented *Chlae1* or *Chvel1*, respectively. Only white pseudothecia (arrows) are produced, indicating the mutant strains are female sterile (color of pseudothecia reflects strain acting as female). Heavy black region (green arrow) on bottom image indicates profuse production of pigmented conidia of the *Chvel1* mutant. **B.** *ChVEL1* overexpression strains are more fertile than WT strains. Crosses with *ChVEL1* OE strains produce both pigmented and white pseudothecia (bottom panel, arrows) at a ratio of 2∶1 while a WT cross produced nearly equal numbers of pigmented and white pseudothecia (top panel). *ChLAE1* OE strains are female sterile (middle panel, white pseudothecia only), like *Chlae1* mutants. **C.** Asexual sporulation is de-repressed in the *Chvel1* mutant during mating. The average numbers of asexual spores formed on cross plates are shown. Error bars are standard deviation. Asterisks indicate p-value <0.05 in T-test analysis in which each strain was compared with WT C4. WT strain C4 and the *Chlae1* mutant strain produce few vegetative spores during sexual reproduction, while asexual sporulation is de-repressed in the *Chvel1* mutant (see also bottom panel in **A,** green arrow).

To determine if ChLae1 and ChVel1 control heterothallic reproductive development, as in homothallic *A. nidulans*, we tested ability of *Chlae1* and *Chvel1* mutants (both *MAT1-2*;*ALB1*) to undergo sexual development by crossing them to CB7, an albino tester strain of opposite mating type (*MAT1-1*;*alb1*). Control crosses of CB7 to WT strain C4 (*MAT1-2*;*ALB1*) produced both pigmented and albino pseudothecia, indicating that both parental strains were hermaphroditic. Crosses involving either the *Chlae1* or *Chvel1* pigmented mutants produced fertile albino pseudothecia, but failed to produce pigmented pseudothecia, indicating that both types of mutant are female sterile, since color of pseudothecia reflects which parent in a cross acted as the female (Figure 5A). Complementation of both *Chlae1* and *Chvel1* mutants restored WT crossing capability (data not shown). This result demonstrates that ChLae1 and ChVel1 have positive roles in sexual differentiation in *C. heterostrophus*, likely in the early stages of fruiting body formation, since no pigmented fruiting bodies were formed. Crosses of *VEL1* OE strains to albino tester CB7 produced both black and white fertile pseudothecia, indicating that OE strains were fully mating competent (Figure 5B). Furthermore, the number of black pseudothecia was at least two times the number of white pseudothecia (data not shown). The ratio between black and albino pseudothecia is usually 1∶1 in crosses between pigmented WT and albino testers. This further confirmed that ChVel1 plays a major role in sexual reproduction. In contrast, when *LAE1* OE strains were crossed to albino tester CB7, they were female sterile (Figure 5B). Thus, the excess of ChLae1, just like its absence, causes loss of ability to act as female, implying that ChLae1 might affect sexual development through influencing gene expression and/or protein levels of other members of the velvet complex, i.e. VelB, VeA, VosA [15].

Asexual sporulation is repressed during sexual development of *C. heterostrophus*; usually very few conidia are found on the corn leaf substrate supporting crosses of WT strains [2]. In contrast, the *Chvel1* mutant produced a large number of conidia on the cross plate substrate, indicating de-repression of asexual spore formation during sexual development. The *Chlae1* mutant was comparable to WT in asexual spore production on cross plates (Figures 5C and 5A, bottom panel, green arrow). Note that the *VEL1* OE strain side of the leaf on cross plates is not heavily pigmented (Figure 5B, bottom panel), in contrast to the *vel1* mutant side of the leaf on cross plates (Figure 5A, bottom panel). The observation indicates that ChVel1 represses asexual sporulation during sexual reproduction.

### ChLae1 and ChVel1 control asexual development during vegetative growth

**Figure 6:**
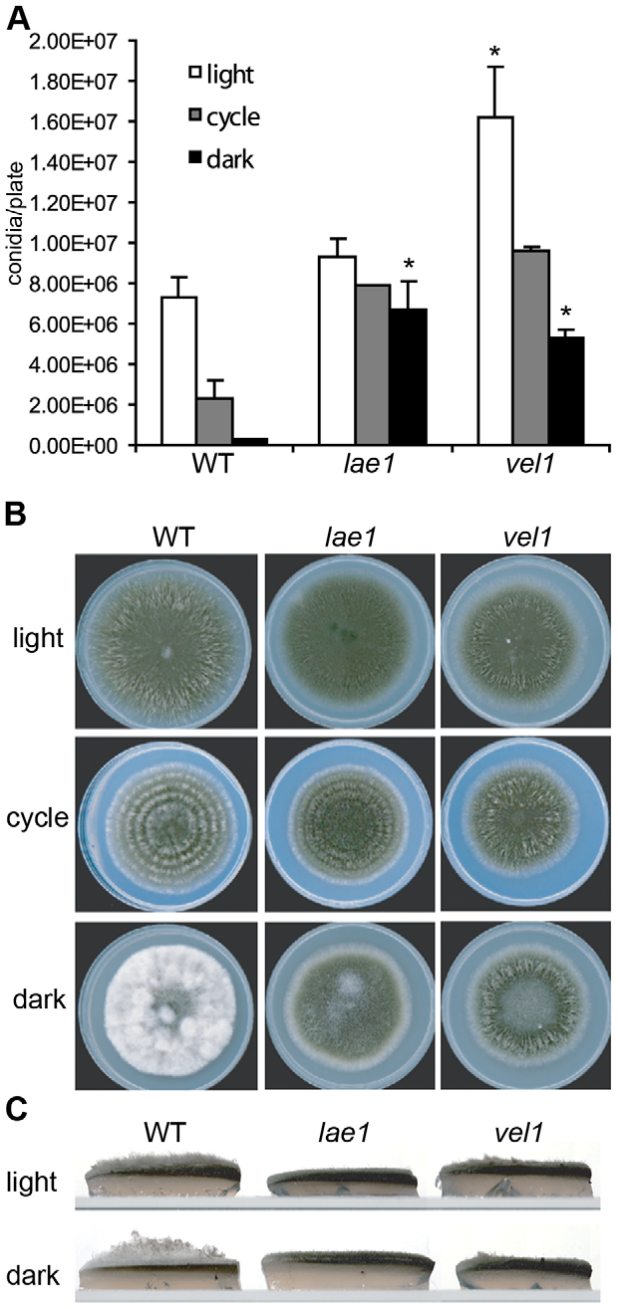
Repression of asexual sporulation in the dark and under cycling conditions is compromised in *Chlae1* and *Chvel1* mutants growing vegetatively. **A.** Quantification of asexual spores from cultures grown under constant light, 12 hour light/dark cycle, and dark conditions. Error bars are standard deviation. Asterisks represent p-value <0.05 in T-test analysis in which each mutant strain was compared with the corresponding WT C4 strain under the same conditions. Asexual sporulation is repressed in WT in the dark or under the light cycling conditions, while this was not observed for the *Chlae1* mutant. Absence of *ChVEL1* augments asexual sporulation regardless of the light condition. **B.** Cultures grown on CMX plates under constant light, 12 hour light/dark cycle and constant dark conditions. Note that in the dark, WT C4 is white and fluffy reflecting aerial hyphal growth and production of very few conidia, while *Chlae1* and *Chvel1* mutants are pigmented. Alternating light and dark conidial banding pattern of the WT strain C4 in middle panel indicates that conidiation of the WT strain is responsive to light. This banding pattern is absent or much reduced in the *Chvel1* mutant, but still evident in the *Chlae1* mutant. **C.** Side view of plates of WT strain C4, and the *Chlae1* and *Chvel1* mutants grown in constant light or dark on CMX. Note aerial hyphae on plates of WT, especially from the dark. In contrast, the surface of the *Chlae1* mutant is very flat while the *Chvel1* mutant shows a small amount of aerial hyphae. Thus Lae1 appears to play a greater role in promoting aerial hyphae growth than Vel1.

To assess the role of ChLae1 and ChVel1 in asexual development during vegetative growth, *Chlae1*, *Chvel1* mutants and WT strains were grown on complete medium with xylose (CMX) as carbon source under constant light, constant dark and 12 hour light/dark cycling conditions for 8 to 10 days, and number of conidia measured. The WT strain produced the most conidia in constant light, very few in constant dark, an intermediate number in cycling conditions (Figure 6A). The *Chlae1* mutant was relieved from this dark-responsive repression and produced numbers of conidia similar to WT in light, under all conditions. Asexual development by the *Chvel1* mutant, however, was significantly increased compared to WT in all light conditions (Figure 6A) as for asexual development on cross plates, described above (Figures 5A, C). Note, however, that the dark-responsive repression of asexual sporulation was still observable for the *Chvel1* mutant, but not for the *Chlae1* mutant. The WT strain had a very clear banding rhythm (reflecting periods of conidiation) under 12 hour light/dark cycling conditions, whereas the *Chvel1* mutant exhibited almost no banding (Figure 6B). WT showed significant development of aerial hyphae in dark, but this was much reduced in the *Chlae1* and *Chvel1* mutants (Figures 6B, C).

Like WT, strains overexpressing either *LAE1* or *VEL1* on PGA medium were white and very fluffy, and produced very few conidia in dark. In cycling, or constant light conditions, *LAE1* OE strains produced statistically fewer conidia than WT, indicating repression by excess Lae1 (Figure S4). Although care should be taken in comparing conidiation on PGA medium (Figure S4), to that on a medium considered optimal for conidium production (CMX, Figures 6A, B), the observation is in line with the hypothesis that ChLae1 negatively regulates asexual sporulation.

Collectively, the data indicate that ChLae1 and ChVel1 control the balance between aerial hyphal growth and conidiation, i.e., they promote aerial hyphal growth and repress conidiation, in response to dark conditions. Similarly, deletion of *F. verticillioides FvVE1* leads to flat hyper-conidiating colonies [49]. Complementation of the *C. heterostrophus* mutants with WT genes restored the WT phenotype (not shown).

### ChLae1 and ChVel1 regulate melanization of mycelia

**Figure 7:**
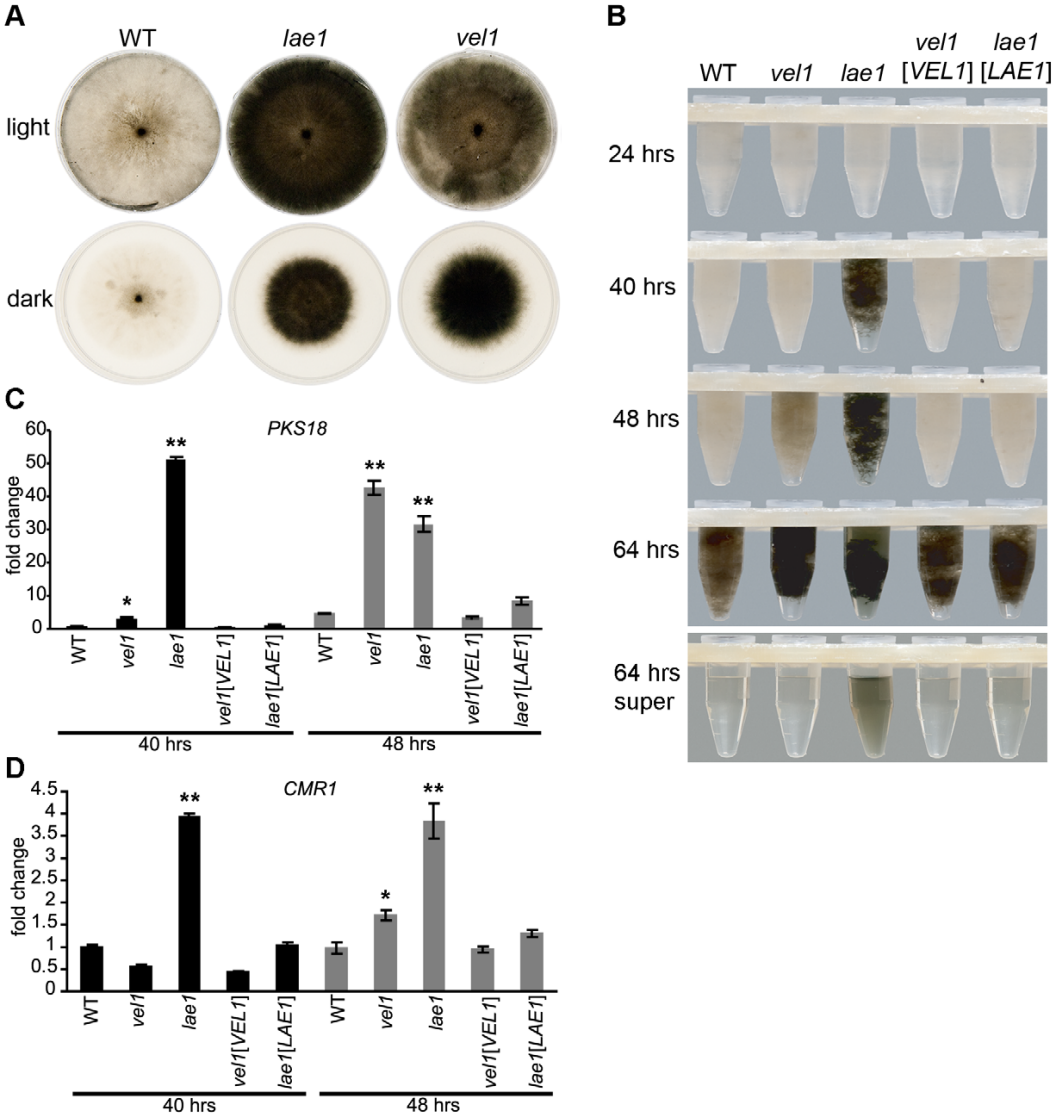
Lae1 and Vel1 proteins negatively regulate mycelial pigmentation of *C. heterostrophus*. **A**. Bottom of culture plates incubated in constant light or dark for 9 days. Pictures were taken after removal of conidia. Note heavy pigmentation of mycelia of *Chlae1* and *Chvel1* mutants in both light and dark compared to WT. **B**. Mycelial pellet of WT strain C4, *Chvel1*, *Chlae1* mutants, and complemented strains (*vel1*[*VEL1*], *lae1*[*LAE1*]) at different time points indicated. Supernatant is shown only for the 64 hour samples. *Chlae1* strain is melanized by 40 hrs, clearly ahead of other strains. Melanization starts by 48 hrs in *Chvel1*, while pigmentation is not evident in WT at this time. The supernatant of the *Chlae1* strain is pigmented indicating secretion of pigment(s) into the medium (no conidia are present). **C**. qPCR analyses of *PKS18*. Expression was examined for the samples from **B** at 40 and 48 hours. Expression level relative to the WT strain C4 sample at 40 hours is shown. Error bars represent range of fold change calculated according to standard deviation of ΔΔCt. Single asterisks indicate p-value <0.05, double asterisks indicate p-value <0.001 in T-test analysis in which each strain was compared with corresponding WT C4 at the same time point. Matching the enhanced pigmentation (**B**), *PKS18* is induced in *Chlae1* by 40 hrs and in *Chvel1* by 48 hrs. **D**. qPCR analyses of *CMR1*. Expression was examined for the samples from **B** at 40 and 48 hours. Expression level relative to the WT, C4 sample at 40 hours is shown. Error bars and T-test analysis are same as **C**. Like the enhanced pigmentation in **B**, *CMR1* expression increased 1.7 and 3.8 fold in *Chvel1* and *Chlae1* mutants, respectively, compared to WT by 48 hrs. Note scale is different from **C**.

Unlike *A. nidulans* and *A. fumigatus* LaeA, deletion of which leads to loss of mycelial pigmentation [12], lack of either ChLae1 or ChVel1 caused increased pigmentation of mycelia grown either on solid (Figure 7A) or in liquid (Figure 7B) medium, in both dark and light, indicating that ChLae1 and ChVel1 negatively regulate mycelial pigmentation in *C. heterostrophus*. Pigment was also evident in the supernatant of the *Chlae1* mutant at 64 hrs (Figure 7B) suggesting that it is secreted into the medium. Hyphal pigmentation develops more slowly in the *Chvel1* mutant than in the *Chlae1* mutant, but faster than in WT. By 64 hrs, all strains were darkly pigmented. Reintroduction of *ChVEL1* or *ChLAE1* restored the WT phenotype (Figure 7B).

To bolster previous evidence that mycelial and conidial melanin of *C. heterostrophus* is the DHN, and not the tyrosine-derived type, we used inhibitors of each (pyroquilon and kojic acid, respectively) to test their effect on melanization. All cultures (WT, *lae1* and *vel1* mutants, and complemented strains) grown on pyroquilon were light brown instead of dark green/black in color, while those grown on kojic acid were unaltered in color (Figure S5), supporting previous data on melanin biosynthesis in *C. heterostrophus* [50], [51]. We tested expression of *PKS18*, the core *PKS*, *CMR1* [51], a transcription factor associated with DHN melanin synthesis, in WT and mutants (Figures 7C, D). Enhanced expression of *PKS18* was observed for both mutants compared to WT. By 40 hrs, *PKS18* expression in *Chvel1* had slightly increased (3 fold), and by 48 hrs, it had increased ∼8 fold over WT, while expression in the *Chlae1* mutant reached its peak (>50 fold) as early as 40 hrs. At 48 hours, *CMR1* expression had increased 1.7 and 3.8 fold in *Chvel1* and *Chlae1* mutants, respectively, compared to WT. For both *PKS18* and *CMR1*, reintroduction of *ChVEL1* or *ChLAE1* restored WT expression levels (Figures 7C, D).

**Figure 8:**
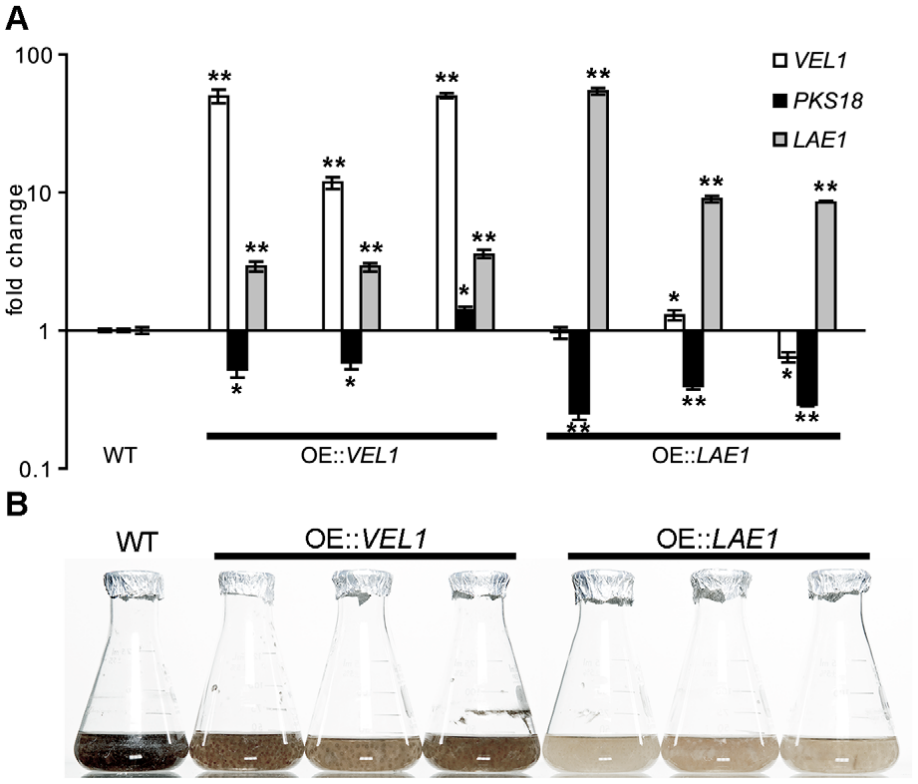
Overexpression of *LAE1* and *VEL1* represses expression of the polyketide synthase gene, *PKS18*, associated with melanin production. **A.** qPCR analyses of *PKS18*, *ChVEL1* and *ChLAE1*. Expression of the genes was determined for the cultures grown for 48 hrs in CM with PGA as the carbon source. WT strain C4, and three independent *ChVEL1* (left to right: OEVEL1-3, OEVEL1-4 and OEVEL1-7) and *ChLAE1* (left to right: OELAE1-1, OELAE1-3 and OELAE1-4) overexpression strains each were examined, and expression level relative to the WT sample at 48 hrs is shown. Error bars represent range of fold change calculated according to standard deviation of ΔΔCt. Single asterisks indicate p-value <0.05, double asterisks indicate p-value <0.001 in T-test analysis in which each strain was compared with WT C4. *ChLAE1* and *ChVEL1* are overexpressed in each OE strains and *PKS18* is repressed in these strains, except for strain OEVEL1-7. The data confirm that ChLae1 and ChVel1 negatively regulate melanin biosynthesis at the transcriptional level. Note that expression of *ChLAE1* is up in *ChVEL1* overexpression strains. **B.** C4, and three independent *ChVEL1* and *ChLAE1* (left to right: as above) overexpression strains each, grown as in **A**. Note that *ChVEL1* and *ChLAE1* overexpression strains displayed less pigmentation than the WT C4 strains with *ChLAE1* OE having the least melanization (compare to *Chlae1* mutant strains Figure 7). This indicates that these proteins are negative regulators of melanization of mycelia and that ChLae1 plays a larger role.

Finally, we tested how overexpression of *LAE1* and *VEL1* affects melanization and *PKS18* expression levels of three independent overexpression strains, grown in PGA liquid culture in the dark. Each overexpression strain exhibited a greater than 10-fold increase in expression level of *VEL1* or *LAE1* compared to WT (Figure 8A). Overexpression strains all showed attenuated mycelial pigmentation (Figure 8B) and decreased *PKS18* expression (Figure 8A), except for one of the OE::*VEL1* strains for which the *PKS18* expression level was slightly higher than in WT. We note that expression of *ChLAE1* was up in OE::*VEL1* strains compared to WT, but expression of *ChVEL1* was not significantly different from WT in OE::*LAE1* strains. Overall, we conclude that both ChLae1 and ChVel1 play a negative role in melanin biosynthesis.

## Discussion

Several secondary metabolites are important for virulence of *C. heterostrophus* to its host maize, including the polyketides, T-toxin and melanin, and the nonribosomal peptide extracellular siderophore [25], [32], [34]. We explored whether or not deletions of *ChVEL1* and *ChLAE1* influence production of these metabolites and found three different outcomes. T-toxin production was negatively impacted and virulence was reduced; melanin production was positively affected; while expression of the *NPS6* gene associated with extracellular siderophore production was not affected, under iron-depleted conditions. Regarding T-toxin, given that the current focus in plant-microbe interaction biology is on effector molecules, and that effectors can be either secondary metabolite HSTs or proteins, our finding opens up the possibility that other types of molecule, such as protein effectors, may be controlled by Lae1/Vel1. We are intrigued by the fact that conserved regulators such as Lae1 and Vel1 influence function of molecules whose associated genes, as well as they, are so astoundingly diverse in structure and in evolutionary history. Genes associated with T-toxin production are a remarkable example, i.e., only two of the nine genes identified to date have phylogenetic profiles that are related, yet all nine are controlled by Lae1 and Vel1 proteins.

Lack of regulation by Lae1/Vel1 of the NPS6 gene associated with extracellular siderophore production is in agreement with a report by Perrin et al. [13] that LaeA is dispensable for the low iron-induced expression of *sidC* and *sidD* in *A. fumigatus*. Thus our observation on *NPS6*, from the maize phytopathogen *C. heterostrophus*, is parallel with their report on *sidD*, the ortholog of *NPS6*, in the human pathogen, *A. fumigatus*. We speculate that this finding reflects the critical roles played by extracellular siderophores of both pathogens in virulence to the host and in fundamental processes of cellular metabolism. Regarding virulence and fundamental cellular metabolism, we also determined that deletion of *ChLAE1* or *ChVEL1* impacts redox status; mutants are reduced in tolerance to H_2_O_2_. Ability to manage ROS is a key determinant of fungal success in the infection court, as well as in basic metabolism of fungal cells. Our study pointed to *CAT3*, one of the three genes encoding the H_2_O_2_ scavenger catalase, as important in the reduced ability of the *Chvel1* mutant to manage oxidative stress.

We also examined the effect of *ChVEL1* and *ChLAE1* deletions on reproductive development and demonstrated that both sexual and asexual development are impacted, thus clearly implicating *C. heterostrophus* Lae1 and Vel1 in regulation of both secondary metabolism and development, as in *Aspergillus* spp. and other fungi.

### 

#### Lae1/Vel1 and T- toxin

T-toxin is an HST effector that confers super-virulence capability on *C. heterostrophus* race T in its interaction with T-cms maize. Nine genes involved in toxin production have been identified, however, their genetic organization is exceedingly complex: the genes are not clustered, they are surrounded by highly repetitive A+T rich sequence, they display disparate phylogenetic signatures [25]–[27], and no regulatory element(s) has been identified. As a step towards understanding key genetic differences and evolutionary mechanisms associated with appearance of highly aggressive pathogens, such as race T, we were prompted to investigate whether *C. heterostrophus* Lae1 and Vel1, corresponding to the *A. nidulans* LaeA and VeA, control HST (T-toxin) production. Given the exceptionally disjointed organization and diverse evolutionary relationships of the *Tox1* genes, we were surprised to find that all known *Tox1* genes are indeed under Lae1 and Vel1 control and are light-regulated. These findings have broad relevance for disease prevention strategies, whether for plants or humans/animals.

In WT, we observed that *Tox1* gene expression was significantly up-shifted when cultures were grown in the dark (Figure 2). ChLae1 and ChVel1 are clearly important for this regulation since *Tox1* gene induction in the dark was minimal or greatly reduced in the corresponding gene deletion strains (Figure 2A) and increased in overexpression strains (Figure 2D). These observations are in line with the results of *E. coli*-based assays to evaluate T-toxin production where a significant reduction (in mutants), or increase (in overexpression strains), in the size of halos, indicative of bacterial death due to T-toxin, was observed compared to WT strains grown in the dark (Figure 1). Overexpression of *VEL1* affected *Tox1* gene expression in the dark only, consistent with translocation of Vel1/VeA into the nucleus in the dark [4], [19]. In contrast, overexpression of *LAE1* resulted in higher *Tox1* gene expression in the light than the dark, indicating lack of the light/dark regulation seen in WT likely due to reduced *VEL1* expression (about 3 fold reduction Figure 2E) in the OE::*LAE1* strains in the dark. Together, these observations demonstrate that ChLae1 and ChVel1 positively regulate T-toxin production in the dark, through transcriptional control of the *Tox1* genes and that stoichiometric levels of Lae1 and Vel1 are key to Lae1/Vel1 function.

Judged by comparative halo sizes (Figure 1A), the ability of WT, grown in the light, to produce T-toxin was greater than abilities of the *Chlae1* and *Chvel1* mutants grown in dark, despite the fact that almost the same (*Chvel1*) or increased levels (*Chlae1*) of *Tox1* gene expression were observed for the mutants compared to WT in the light. Along with the much earlier work which demonstrated that light facilitates T-toxin activity [52], [53], the observation implies that an additional regulatory mechanism for T-toxin production, possibly at the posttranslational level, is operational when the fungus is grown in light. The reduced T-toxin production of the *Chlae1* mutant compared to WT in light indicates that ChLae1 is required for the light-responsive stimulation of toxin synthesis. An additional, unknown, regulatory mechanism was implicated when T-toxin production was evaluated for different parts (i.e. different ages) of the WT culture grown in dark (Figure 1A). Increased T-toxin production was observed at the edge of the colony compared to the center, suggesting an age-dependent decrease in T-toxin production. This was not observed for WT cultures grown in light. The age-dependent fluctuation of toxin production is absent in the *Chlae1* mutant grown in dark, while it is still detectable in *Chvel1* mutant. Curiously, a minor increase in T-toxin production in younger parts of the culture compared to older (except the oldest at the colony center) was observed for the *Chvel1* mutant even when grown in light. These findings indicate that *Ch*Lae1 is involved in the age-dependent regulation of T-toxin production in dark, and that *Ch*Vel1 counteracts the age-dependent regulation in light.

Altogether, our observations indicate that there are at least three different regulatory mechanisms for T-toxin production. In *A. nidulans*, LaeA plays regulatory roles through interactions not only with VeA but also additional components. As discussed below, this could also be the case in *C. heterostrophus*. ChLae1, most likely in the form of a ChLae1-ChVel1 complex, transcriptionally induces T-toxin production in dark. On the other hand, it is conceivable that ChLae1 forms a complex with component(s) other than ChVel1 for the light-responsive stimulation and age-dependent regulation of the toxin synthesis. ChVel1 may be indirectly involved in the second and third regulatory systems by competing for ChLae1 with other components.

*Chlae1* and *Chvel1* mutants showed reduced virulence to both T- and N- cytoplasm maize. Spores from both types of mutant germinated and made normal appressoria (data not shown), but were unable to colonize as extensively as WT. For comparison, we included an extracellular siderophore mutant (*nps6*) in our experiments, as it is much reduced in virulence, hypersensitive to ROS and low iron, but still makes T-toxin. On both N- and T-cytoplasm plants, the *nps6* mutant developed the most reduced size of lesions, followed next by the *lae1*, then the *vel1* mutant (Figure 3). In terms of chlorosis conferred by T-toxin, however, the *nps6* mutant was comparable to WT (Figure 3), whereas the *lae1* and *vel1* mutants were clearly reduced in amount of chlorosis, indicating that Lae1 and Vel1 control high virulence due to production of the secondary metabolite, T-toxin, in addition to basic pathogenicity (lesion formation), *in planta*.

#### Lae1/Vel1 and ROS sensitivity

In aerobic environments, fungi face detrimental effects of ROS generated from environmental and biological sources (e.g. defense responses by host organisms) and have developed multiple ways to manage this stress. For *C. heterostrophus*, the transcription factor ChAp1, an oxidative stress sensor, induces expression of genes associated with oxidative stress management, such as those encoding thioredoxin (*TRX2*), thioredoxin reductase (*TRR1*) and glutathione synthetase (*GSH2*) [47]. Other enzymes such as superoxide dismutases, catalases, and peroxidases also contribute to fungal tolerance to ROS. Regulation of oxidative stress response in *C. heterostrophus* is known to involve the Hog1 MAPK pathway [54] and the histidine kinase response regulators, Ssk1 and Skn7 [2], as well as the nonribosomal peptide extracellular siderophore involved in iron metabolism. Deletion of *ChLAE1* and *ChVEL1* compromised tolerance to H_2_O_2_. In our search for possible ChLae1 and ChVel1 regulons, we characterized expression of most known *C. heterostrophus* oxidative stress response genes in *Chlae1* and *Chvel1* mutants. For the *Chlae1* mutant, none of the genes examined was significantly affected in expression, when compared to WT. For the *Chvel1* mutant, dramatic reduction in *CAT3* expression level and a slight reduction in *GSH2* and *GRX6* expression levels were observed when compared to WT, indicating some of the known oxidative stress response genes are under the control of ChVel1. Further work is required to elucidate the molecular mechanism(s) accounting for the increased sensitivity of the *Chlae1* and *Chvel1* mutants to ROS. To our knowledge, this is the first report that a VeA ortholog controls expression of genes associated with oxidative stress tolerance. Bayram *et al.* found veA mutants are slightly sensitive to H_2_O_2_, which they attributed to trehalose reduction [15].

#### Lae1/Vel1 and melanin

Although virulence of a *C. heterostrophus* albino strain is indistinguishable from that of a pigmented strain under laboratory conditions, lack of pigment compromises fitness of the fungus under natural conditions, and consequently an albino strain cannot survive in the field [34]. In this study, we found that deletion of *ChLAE1* or *ChVEL1* leads to increased mycelial pigmentation in *C. heterostrophus*, indicating that the corresponding proteins negatively regulate pigmentation. Changes in mycelial pigmentation have been reported for *laeA* and *veA* mutants of other species. Pigmentation of *laeA/lae1* mutants was reduced or absent in *F. fujikuroi*, *A. nidulans*, *A. fumigatus*, and *P. chrysogenum* [5], [8], [12]. VeA/Vel1 negatively regulates mycelial pigmentation in *F. fujikuroi* [8], however it plays a positive role in melanization in *Mycosphaerella graminicola*, a dothideomycete relative of *C. heterostrophus* [41]. Apparently, LaeA and VeA orthologs can influence pigmentation positively or negatively in a species-specific manner.

We note that expression of *ChLAE1* was elevated in *VEL1* OE strains compared to WT, but expression of *ChVEL1* was not significantly different from WT in *ChLAE1* OE strains. We speculate that ChLae1 plays a major role in melanin biosynthesis regulation, while ChVel1 affects melanin production through regulating *ChLAE1* expression. This theory is consistent with the observation that pigmentation was dramatically reduced in *ChLAE1* OE strains but expression of *ChVEL1* was not changed, whereas pigmentation was moderately reduced and expression of *ChLAE1* was modestly increased in *ChVEL1* OE strains. This upregulation of *LAE1* in *VEL1* OE strains is different from the study of Amaike et. al. with *A. flavus* where overexpression of *VEA* downregulated *LAEA* mRNA levels [55].

#### Lae1/Vel1 and reproduction

VeA has been defined as an inhibitor of asexual development and key player in sexual development in filamentous fungi [4], [9], with some exceptions in *Aspergillus parasiticus* [56] and *F. fujikuroi* [8]. In our work, deletion of *ChVEL1* resulted in increased asexual reproduction under all conditions tested and inability to mate as female, while overexpression of *ChVEL1* resulted in strains that were super-fertile as females, demonstrating a negative role in asexual and a positive role in sexual development. Like ChVel1, *A. nidulans* VeA positively regulates sexual reproduction [9], but *F. fujikuroi* Vel1 apparently controls sexual differentiation in the opposite way, given that an increased number of perithecia developed when *vel1* mutants were mated [8]. With regard to asexual development, VeA homologs can also play different roles in different fungal species. For example, in *C. heterostrophus* (this work), *A. nidulans*, *F. verticillioides*, and *Neurospora crassa*, VeA/Vel1 plays a negative role in asexual differentiation [49], [57], [58], whereas, in *A. flavus* (morphotype dependent), *P. chrysogenum* and *F. graminearum*, it positively regulates asexual reproduction [11], [55], [59]. Due to choice of strain background (*veA1*), *A. nidulans* LaeA was first suggested as a secondary metabolism regulator only [12]. However, recent evidence indicates that LaeA also plays important roles in fungal reproduction. Asexual sporulation is reduced in *laeA* mutants of *A. nidulans* [15], *P. chrysogenum* [5], *F. fujikuroi* [8]. In contrast, ChLae1 plays a negative role in asexual development during the vegetative phase in *C. heterostrophus*, as does ChVel1, as conidiation is increased in mutants, similar to *A. flavus laeA* mutants under low inoculum conditions [55]. *A. nidulans* LaeA inhibits sexual development in the light and regulates Hülle cell formation [15]. Our data indicate that ChLae1 is essential for female fertility in *C. heterostrophus* as both mutants and OE strains were female sterile. This differs from studies with *A. flavus* LaeA in which loss of *LaeA* results in no sclerotia while LaeA OE strains overproduce sclerotia [55]. The OE phenotype of *Chlae1* mutants is likely due to altered *ChVEL1* expression levels in *ChLAE1* OE strains. We observed upregulation of *ChVEL1* in *Chlae1* null mutant under vegetative growth condition (data not shown), therefore, OE of *ChLAE1* could inhibit expression of *ChVEL1* on cross media, leading to loss of female sterility. We observed no significant changes of *ChVEL1* expression in *LAE1* OE strains in liquid culture (Figure 8A), but 3 fold reduction on solid medium (Figure 2E). It will be interesting to check if expression of *ChVEL1* is altered in the *ChLAE1* OE strain on cross plates.

As shown in this study and those of others, LaeA/Lae1 and VeA/Vel1 play roles both in development and secondary metabolism. However, deletion or knockdown of each of these genes can cause similar but also different phenotypes, implying that LaeA/Lae1 and VeA/Vel1 act independently, as well as in collaboration with each other. A clue to this is found in a recent report by Bayram *et. al.* 2010 [15] indicating that an additional member of the velvet complex, VelB, forms a second complex with VosA to repress asexual development in *A. nidulans*. LaeA regulates both velvet (VelB-VeA-LaeA) and VelB-VosA complex formation. This multi-complex interplay provides LaeA/Lae1 and VeA/Vel1 with both similar and different roles. VelB and VosA homologs are detectable in the *C. heterostrophus* genome.

In this study, both ChLae1 and ChVel1 repress asexual sporulation in dark, however, the mutants showed different phenotypes in the light. Deletion of *ChLAE1* did not affect sporulation in light, whereas the *Chvel1* mutant showed 2-fold increase in asexual sporulation compared to WT. A similar phenotypic discrepancy was observed for the *A. nidulans laeA*- and *veA*-deletion strains [15]. The *laeA* and *veA* mutants were indistinguishable in terms of asexual sporulation in dark, however, the *veA* mutant produced approximately twice as many spores as the *laeA* mutant did in light. The *A. nidulans veA laeA* double mutant showed *veA*-like sporulation in light, indicating that VeA is epistatic to LaeA with respect to the asexual sporulation phenotype. Deletion of *LaeA* promotes formation of the VelB-VosA complex, which negatively regulates asexual sporulation, in light. On the other hand, VelB is preferentially imported to nuclei in the form of a VelB-VeA complex, and thus deletion of *VeA* can indirectly repress VelB-VosA heterodimer formation. Therefore, the VelB-VosA complex may account for the difference in asexual sporulation in light between the *laeA*- and *veA*-deletion mutants. The situation could be more complicated in *C. heterostrophus*, given that asexual sporulation in light is enhanced in the *Chvel1* mutant compared to WT. By analogy to *A. nidulans*, lack of ChLaeA and ChVel1 is likely to affect levels and interactions with other proteins. It will be a challenging task to link the phenotypes of the *Chlae1* and *Chvel1* mutants to the true functions of the presumed ChLae1-Vel1-VelB complex.

## Materials and Methods

### Fungal strains, plant materials, and general growth conditions

*C. heterostrophus* strains C4 (*Tox1* ^+^;*MAT1-2*, ATCC 48331), C5 (*Tox1* ^−^;*MAT1-1*, ATCC 48332) [60] and Chnps6-3 (*NPS6* deletion) [32] were used. *ChLAE1* (strain ChW5) and *ChVEL1* (strain ChW7) gene-deletion strains were generated in the C4 genomic background, as was Chnps6-3. ChW4 (*vel1*[*VEL1*]) and ChW6 (*lae1*[*LAE1*]) are strains complemented with the WT *VEL1* and *LAE1* genes, respectively (Table 1). Unless mentioned otherwise, all strains were grown on CMX [27] under 16 hr fluorescent light at approximately 22°C.

**Table 1:** *Cochliobolus heterostrophus* strains used in this study.

Strain	Genotype	Comment(s)
C4	*MAT1-2 Tox1+*	WT, ATCC 48331
C5	*MAT1-1 tox1−*	WT, ATCC 48332
CB7	*MAT1-1 alb1*	WT, B30-A3-R-20, albino
Chnps6-3	*MAT1-2 Tox1+ nps6 hygB^R^*	C4 background, full-length deletion
ChW7	*MAT1-2 vel1 hygB^R^*	C4 background, full-length deletion
ChW4-1	*MAT1-2 vel1*[*VEL1*] *NPTII*	ChW7 complemented
ChW4-3	*MAT1-2 vel1*[*VEL1*] *NPTII*	ChW7 complemented
ChW5	*MAT1-2 lae1 hygB^R^*	C4 background, full-length deletion
ChW6-1	*MAT1-2 lae1*[*LEA1*] *NPTII*	ChW5 complemented
ChW6-3	*MAT1-2 lae1*[*LEA1*] *NPTII*	ChW5 complemented
ChW6-10	*MAT1-2 lae1*[*LEA1*] *NPTII*	ChW5 complemented
ChW6-18	*MAT1-2 lae1*[*LEA1*] *NPTII*	ChW5 complemented
OEVEL1-3	*MAT1-2 OE::VEL1 hygB^R^*	C4 background, with additional copy of *VEL1* driven by pelA promoter
OEVEL1-4	*MAT1-2 OE::VEL1 hygB^R^*	C4 background, with additional copy of *VEL1* driven by pelA promoter
OEVEL1-7	*MAT1-2 OE::VEL1 hygB^R^*	C4 background, with additional copy of *VEL1* driven by pelA promoter
OELAE1-1	*MAT1-2 OE::LAE1 hygB^R^*	C4 background, with additional copy of *LAE1* driven by pelA promoter
OELAE1-3	*MAT1-2 OE::LAE1 hygB^R^*	C4 background, with additional copy of *LAE1* driven by pelA promoter
OELAE1-4	*MAT1-2 OE::LAE1 hygB^R^*	C4 background, with additional copy of *LAE1* driven by pelA promoter

*Zea mays* cultivars W64A-N and W64A-T were grown in a growth chamber under 16 hours of light/8 hours of dark at 24°C as previously described [32].

### Identification of LaeA and VeA orthologs in *C. heterostrophus*

The *A. nidulans* LaeA (accession number: AAQ95166) and VeA (accession number: AAD42946) genes were used to query the *C. heterostrophus* strain C5 sequence database (http://genome.jgi-psf.org/CocheC5_1/CocheC5_1.home.html) for orthologs. Alignments were created using ClustalW and phylogenetic trees built using PAUP 4.0.

### Gene deletion, PCR confirmation and complementation

Both *ChLAE1* and *ChVEL1* were deleted using the split marker method [61] and transformation protocol described previously [62]. Integration at the target sites was confirmed as described in [27]. The *Chvel1* mutant was complemented using the co-transformation method described in [63] with a minor modification. The *nptII* carrying plasmid, pNG, which carries *nptII*, instead of the *bar* (for bialaphos resistance) in plasmid pBG [64], at the *Bam*HI cloning site, was linearized with *Bst*BI before use in co-transformation with the *ChVEL1* coding and flanking sequences. The *Chlae1* mutant was complemented using direct transformation after unsuccessful attempts at co-transformation. For this, the *nptII* gene amplified from pNG was stitched to the 3′ end of the 3′ *LAE1* flanking sequence by overlapping PCR. This construct was then linked to a sequence further downstream of the 3′ *LAE1* flanking sequence. The final construct carried both the WT *LAE1* gene and the *nptII* selectable marker, plus *LAE1* flanking sequences for targeted integration at the original locus (Figure S6). Sequences of primers for gene deletion, PCR confirmation and complementation, are listed in Table S1.

### Construction of gene overexpression strains

Coding sequences of *ChLAE1* and *ChVEL1* were amplified from genomic DNA of strain C4 with primers tailed with *Bam*HI and *Hin*dIII recognition sites, respectively. The resulting PCR products were digested with the corresponding restriction enzymes then ligated to vector pHNU3PelA [65] precut with both *Bam*HI and *Hin*dIII (Figure S7). pHNU3PelA contains the *pelA* promoter from *A nidulans* which is inducible by polygalacturonic acid (PGA) and repressible by glucose [65]. The constructs, named pHPVEL1 and pHPLAE1, were transformed into WT strain C4 as described [62]. PCR confirmed that both plasmids integrated by single crossover at their corresponding native loci (i.e. pHPVEL1 at *ChVEL1* and pHPLAE1 at *ChLAE1*) (Figure S7), thus each carried two copies of the *LAE1* or *VEL1* gene, one driven by the native promoter, and the other by the inducible PGA promoter. Primer sequences for plasmid construction and verification of integration are in Table S1. Three *ChVEL1* (OEVEL1-3, OEVEL1-4 and OEVEL1-7) and three *ChLAE1* (OELAE1-1, OELAE1-3 and OELAE1-4) overexpression strains were examined.

### Fungal RNA extraction and expression analyses

Total RNAs were isolated from mycelia grown either in liquid medium (CM) or on agar plates (CMX) [60], using the RNeasy Plant mini kit (Qiagen) and then treated with components of the Ambion TURBO DNA-free kit (Applied Biosystems) to remove genomic DNA. cDNA was synthesized from the extracted RNAs using the SuperScript III First-Strand cDNA Synthesis System (Invitrogen). RT- PCR was done with GoTaq DNA polymerase (Promega). qPCR was performed on an ABI Prism 7000 Sequence Detection System with SYBR Green PCR Master Mix (Applied Biosystems). Results were analyzed with the comparative C_T_ method (ΔΔCt) [66]. For RT-PCR, 24 cycles were used for PCR of the internal control actin-encoding gene (*ACT1*) and 27 cycles were used for PCR of T-toxin target genes.

### Sexual and asexual reproduction

Sexual and asexual reproduction measurements were performed as described [2].

### Oxidative stress sensitivity assays

Assays of sensitivity to H_2_O_2_ and induction of genes associated with oxidative stress were as described [2], [47]. 4, 2 and 1 ul of conidial suspension (≈50 spore/ul) of *Chlae1*, *Chvel1*, *nps6* mutants, WT strain C4, *vel1*[*ChVEL1*] strain ChW4, and *lae1*[*ChLAE1*] strain ChW6 complemented strains were inoculated on CM and CM supplemented with 2 or 4 mM H_2_O_2_ and grown for 3 days in dark at 30°C. For expression analyses, the same strains were grown in liquid CM for 40 hours at room temperature (time 0). Samples were collected for RNA extraction at time 0 and at 30 minutes after addition of H_2_O_2_ at a final concentration of 20 mM.

### T-toxin bacterial assay

The T-toxin microbial plate assays were done as described [27], [45]. All assays were done with 8 day old cultures incubated at 19°C.

### Virulence

Virulence of *C. heterostrophus* was evaluated as described previously [32]. Three week-old corn plants (cultivar W64-A N and T cytoplasm) were inoculated with 2 ml conidial suspension (∼5×10^3^/ml) per plant. For overexpression experiments, the spore suspension was supplemented with PGA. For each fungal strain, at least four replicates (*i.e.* inoculation of four independent plants) were used and experiments were repeated three times. Photographed leaves were imaged in Photoshop CS5 and lesion length (chlorosis and necrosis) was measured with a ruler. To measure the symptom caused by T-toxin on T-cms corn leaves, the ratio of length of chlorosis over length of necrosis was used. Statistical analysis was done by T-test.

### Pigmentation

To examine pigmentation of hyphae on solid medium, WT C4, *Chlae1*, and *Chvel1* mutants were grown on CMX under constant light or dark conditions for 9 days. Colony surfaces were scraped and conidia washed off and photographed. To examine pigmentation in liquid medium, WT C4, *Chlae1*, *Chvel1* mutants and their corresponding complemented strains (ChW4 for *ChVEL1* complementation, ChW6 for *ChLAE1* complementation) were grown in liquid CM in dark and 1.5 ml of culture was transferred to eppendorf tubes at 24, 40, 48, and 64 hours and spun down. Supernatants were transferred to new tubes, and they and the pellet fractions photographed. Samples at 40 hours and 48 hours were used for qPCR analyses of *PKS18* and *CMR1*. Gene expression level was expressed as fold change versus that of WT C4 at 40 hours.

For overexpression studies, WT C4, *ChVEL1* and *ChLAE1* overexpression strains were grown in 100 ml minimal medium in 250 ml flasks with PGA as a carbon source for 48 hrs in dark. qPCR of *PKS18*, *ChVEL1* and *ChLAE1* was performed as described above. Value of WT C4 at 48 hours was set as 1.

For characterizing melanin types, two different melanin inhibitors, pyroquilon (a DHN type melanin inhibitor which blocks the conversion of 1,3,8-THN to vermelone) and kojic acid (which blocks tyrosinase activity) [67], [68], were added to the final concentration of 10 ug/ml and 100 ug/ml, respectively, in CMX medium. WT C4, *Chlae1*, *Chvel1* mutants and their corresponding complemented strains were grow on CMX, pyroquilon and kojic acid containing media under constant light for 7 days. Plates were photographed before and after colony surfaces were scraped and conidia washed off.

### GenBank Accession numbers

AcLaeA: XP_001268793; AdLaeA: EEQ74217; AgLaeA: EFQ98498; AnLaeA: AAQ95166); AfLaeA: AAR01218; AflLaeA: AAX68412; AoLaeA: XP_001819665; ApLaeA: AAX68415; AsLaeA: AAX68413; CpLaeA: EER26754; LbLaeA: EDR14855; PbLaeA: EEH15895; PchLaeA: ACD50375; FfLae1: CBE54370; *ChLAE1*: JF826792;MpLaeA: ABA87010; MpeLaeA: EEB93804; NfLaeA: XP_001264291; PcLaeA: ADL63139;PmLaeA: EEA26362; ScLaeA: EFJ01286; TsLaeA: EED22239; UrLaeA: EEP82232; VaLaeA: EEY21263; AbeVeA: EFE31515; AchVeA: CAL68582; AcVeA: EAW07578; AdVeA: EEQ77180; AfVeA: CAE47975; AflVeA: ABC41691; AnVeA: AAD42946; AniVeA: EHA18105; ApVeA: AAS07022; *ChVEL1*: JF826791; FfVel1: CBE54373; FgVe1: HQ436464; FmVe1: ABC02879; NcVeA: EAA27918; NfVeA: EAW22616; PbVeA: EEH41507; PchVeA: CAP92389; PmVeA: EEA25748; ScVeA: EFI93289; TsVeA: EED21639; TvVeA: EFE45170; VelB: ABQ17967; VosA: ABI51618; RodA: AAB60712; Pks1: AAB08104; Pks2: ABB76806; Lam1: ACP43390; Dec1: AAM88291; Red1: AAM88292; Red2: ACP34152; Red3: ACP34153; Tox9: ADB23431; Oxi1: ADB23430; Nps6: AAX09988; *ChAP1*: AY486156; *CAT1*: AY369262; *CAT2*: AY369263; *CAT3*: AY369264; *SKN7*: AY456028; *SSK1*: HM152026; MAT1-1: CAA48465; MAT1-2: CAA48464; *PKS18*: AY495659; *CMR1*: DQ902714; ChK1: AAF05913; Cga1: AAC23576; Cgb1: AAO25585;LlmF: XP_664353.

### JGI model names (http://genome.jgi-psf.org/pages/search-for-genes.jsf?organism=dothideomycetes)

AbLaeA: AB03558.1; Ch112516: estExt_Genewise1Plus.C_230028; Ch19119: fgenesh1_pg.C_scaffold_7000105; Ch24542: estExt_fgenesh1_kg.C_10021; Ch26577: estExt_fgenesh1_kg.C_430002; Ch28900: estExt_fgenesh1_pg.C_50334; Ch39571: gw1.10.215.1; Ch64638: e_gw1.7.1035.1; DsLaeA: e_gw1.1.991.1; HpLaeA: NODE_244_length_406169_cov_26..g3563.t1; MfLaeA: estExt_fgenesh1_kg.C_20358;MgLaeA: estExt_fgenesh1_pg.C_chr_10913; PtLaeA: PTRT_04504; RrLaeA: NODE_25109_length_74158_cov_41.g11943.t1; SmLaeA: estExt_fgenesh1_kg.C_3_t20075; SnLaeA: SNOG_11365.3; StLaeA: fgenesh1_pm.1_#_110;

AbVeA: AB08060.1; DsVeA: estExt_fgenesh1_kg.C_2_t20018; HpVeA: NODE_50_length_318014_cov_25.9.g981.t1; LbVeA: gm1.12491_g; MVe1: e_gw1.2.779.1; MfVeA: Mycfi1.estExt_fgenesh1_pg.C_140014; MgVeA: e_gw1.2.779.1; PtVeA: PTRT_03646; RrVeA: NODE_929_length_97559_cov_41.6.g4671.t1; SmVeA: estExt_fgenesh1_kg.C_2_t20128; StVeA: estExt_fgenesh1_pm.C_220032; ChGsh2: estExt_Genewise1.C_7_t50456; Trx1: estExt_Genemark1.C_170072; Trx2: estExt_Genewise1Plus.C_17_t20462; Trr1: fgenesh1_pm.4_#_575.

## Supporting Information

Figure S1 *C. heterostrophus* Lae1 is an ortholog of *A. nidulans* LaeA. **A.** Phylogenetic analysis. Protein sequences were obtained from each corresponding JGI genome database (http://genome.jgi-psf.org/dothideomycetes/dothideomycetes.info.html) using *Aspergillus nidulans* (An) LaeA as a query: Dothideomycetes: *Alternaria brassicicola (Ab)*, *Mycosphaerella graminicola (Mg)*, *Stagonospora nodorum (Sn)*, *Cochliobolus heterostrophus (Ch)*, *Dothistroma septosporum (Ds)*, *Hysterium pulicare (Hp)*, *Mycosphaerella fijiensis (Mf)*, *Pyrenophora tritici-repentis* (*Pt*), *Rhytidhysteron rufulum (Rr)*, *Septoria musiva (Sm)*, *Setosphaeria turcica (St)*, Basidiomycetes: *Laccaria bicolor (Lb)*, *Schizophyllum commune (Sc)*. Additional protein sequences are from GenBank. Eurotiomycetes: *Ajellomyces dermatitidis (Ad)*, *Arthroderma benhamiae (Abe)*, *Arthroderma gypseum (Ag)*, *Aspergillus clavatus (Ac)*, *Aspergillus fumigatus (Af)*, *Aspergillus flavus (Afl)*, *Aspergillus nidulans (An)*, *Aspergillus niger (Ani)*, *Aspergillus oryzae (Ao)*, *Aspergillus parasiticus (Ap)*, *Aspergillus sojae (As)*, *Coccidioides posadasii (Cp)*, *Monascus pilosus (Mp)*, *Neosartorya fischeri (Nf)*, *Paracoccidioides brasiliensis (Pb)*, *Penicillium citrinum (Pc)*, *Penicillium chrysogenum (Pch)*, *Penicillium marneffei (Pm)*, *Talaromyces stipitatus (Ts)*, *Trichophyton verrucosum (Tv)*, *Uncinocarpus reesii (Ur)*. Sordariomycetes: *Acremonium chrysogenum (Ach)*, *Fusarium fujikuroi (Ff)*, *Fusarium moniliformis (Fm)*, *Neurospora crassa (Nc)*, *Verticillium albo-atrum (Va)*. Basidiomycetes: *Moniliophthora perniciosa (Mpe)*. AnLaeA, ChLae1, and AnLlmF are boxed and highlighted in yellow. **B.** ChLae1/LaeA amino acid alignments of ChLae1, AfLaeA and AnLaeA proteins. *A. fumigatus* (Accession AAR01218, Af-LaeA 349 amino acids), *A. nidulans* (Accession AAQ95166, AnLaeA, 374 amino acids), and *C. heterostrophus* (Accession JF826792, ChLae1, 292 amino acids) were aligned using ClustalW. Conserved S-adenosylmethionine binding sites are marked in red highlights, Asterisks indicate identical residues, colons indicate conserved residues, and periods mark semi-conserved residues. ChLae1 groups with single candidate LaeA orthologous proteins in a well-supported group (arrow) of proteins (blue oval shadow) that is sister to the Eurotiomycete group into which AnLaeA falls. Other putative *C. heterostrophus* SAM binding proteins, identified when the *C. heterostrophus* genome database was queried with AnLaeA, fall in a separate less well-supported group that also includes AnLlmF [42].
(PDF)

Figure S2 *C. heterostrophus* Vel1 is an ortholog of *A. nidulans* VeA. **A.** Methods and species used as in Figure S1A. The single candidate ortholog, ChVel1, groups with single candidate VeA orthologous proteins in a well-supported group of proteins (blue oval shadow) that is sister to the Eurotiomycete group into which AnVeA falls. AnVeA and ChVel1 are boxed and highlighted in yellow. **B.** Amino acid alignment of ChVel1, AfVeA, and AnVeA proteins. *A. fumigatus* (Accession CAE47975, Af-VeA, 570 amino acids), *A. nidulans* (Accession AAD42946, AnVeA, 573 amino acids), and *C. heterostrophus* (Accession JF826791, ChVel1, 593 amino acids) were aligned using ClustalW. Putative NLS predicted by Wolf PSORT [43] is highlighted in purple. Potential α importin-dependent monopartite NLS by cNLS Mapper [44] is in blue. Potential PEST domains predicted by EMBOSS ‘epestfind’ are marked in green. Asterisks, colons, and periods as in Figure S1B.
(PDF)

Figure S3 *ChLAE1* overexpression strains produce more T-toxin. Plugs of each strain (OE::*LAE1*, WT race T strain C4) were grown on minimal medium with glucose, polygalacturonic acid (PGA) or xylose+PGA as the carbon source, in the light. Clear area (halo) indicates T-toxin production and killing of *E. coli* cells. Gridded paper was placed under the plates to help visualize the inner very clear area of the halo. The bottom single plug is race O, T-toxin^−^ strain C5 control (no halo). Left two columns are two replicates of *ChLAE1* overexpression strain OELaeA-1. Right two columns are replicates of race T, strain C4. In this example, the *ChLAE1* overexpression strain makes more T-toxin than WT (compare two plugs from the OE strain to two from WT, second row from top), due to enhanced expression under PGA induction. Note that the *ChLAE1* overexpression strain contains two copies of *ChLEA1*, but that, in the presence of glucose, there is less toxin than when PGA is present (compare two plugs from the OE strain, second row from top to two plugs from the OE strain, top row).
(TIF)

Figure S4 Overexpression of *ChLAE1* and *ChVEL1* alters asexual development. Asexual sporulation is repressed in *ChLAE1* OE strains. The average number of asexual spores formed on PGA plates in constant light and in cycling conditions are shown. Error bars are standard deviation. Asterisks indicate p-value<0.05 in T-test analysis in which each strain was compared with WT C4 under the same light condition.
(TIF)

Figure S5 *C. heterostrophus* produces DHN-type melanin. **A.** Cultures grown on CMX and CMX containing pyroquilon or kojic acid under constant light for 7 days. Addition of pyroquilon altered the pigmentation of conidia and hyphae from dark green to light brown, while kojic acid had no effect on pigmentation. **B**. Culture plates from **A** after removal of conidia. Mycelial color was light brown for all strains tested on pyroquilon medium but unchanged on kojic acid plates.
(TIF)

Figure S6 Strategy to complement the *Chlae1* mutant with WT *ChLAE1*. The construct, described in Materials and Methods, was transformed into *Chlae1* mutant ChW5. A double crossover homologous recombination event would replace the *hygB^R^* marker with the WT *ChLAE1* gene and the *NPTII* marker (G418 resistant) at the *ChLae1* locus. The resulting strains are hygromycin B sensitive and G418 resistant.
(TIF)

Figure S7 Strategy to overexpress *ChLAE1* and *ChVEL1*. Plasmid containing either the *ChLAE1* or *ChVEL1* coding sequence driven by the *pelA* promoter (inducible by polyglacturonic acid, PGA) was transformed into WT strain C4. By a single crossover homologous integration event, a copy of PGA-inducible *ChLAE1* or *ChVEL1* was inserted adjacent to a copy of the same gene driven by its endogenous promoter. After induction by PGA, strains should express both the WT and introduced copies of the *ChLAE1* or *ChVEL1* genes.
(TIF)

Table S1 Primers used for this study.
(DOC)
