# Supplementary material for: ChLae1 and ChVel1 Regulate T-toxin Production, Virulence, Oxidative Stress Response, and Development of the Maize Pathogen Cochliobolus heterostrophus
Source: PLoS Pathog. 2012 Feb 23;8(2):e1002542. doi: 10.1371/journal.ppat.1002542 (PMC3285592; doi:10.1371/journal.ppat.1002542)
Supplement: Figure S1 — C. heterostrophus Lae1 is an ortholog of A. nidulans LaeA. A. Phylogenetic analysis. Protein sequences were obtained from each corresponding JGI genome database (http://genome.jgi-psf.org/dothideomycetes/dothideomycetes.info.html) using Aspergillus nidulans (An) LaeA as a query: Dothideomycetes: Alternaria brassicicola (Ab), Mycosphaerella graminicola (Mg), Stagonospora nodorum (Sn), Cochliobolus heterostrophus (Ch), Dothistroma septosporum (Ds), Hysterium pulicare (Hp), Mycosphaerella fijiensis (Mf), Pyrenophora tritici-repentis (Pt), Rhytidhysteron rufulum (Rr), Septoria musiva (Sm), Setosphaeria turcica (St), Basidiomycetes: Laccaria bicolor (Lb), Schizophyllum commune (Sc). Additional protein sequences are from GenBank. Eurotiomycetes: Ajellomyces dermatitidis (Ad), Arthroderma benhamiae (Abe), Arthroderma gypseum (Ag), Aspergillus clavatus (Ac), Aspergillus fumigatus (Af), Aspergillus flavus (Afl), Aspergillus nidulans (An), Aspergillus niger (Ani), Aspergillus oryzae (Ao), Aspergillus parasiticus (Ap), Aspergillus sojae (As), Coccidioides posadasii (Cp), Monascus pilosus (Mp), Neosartorya fischeri (Nf), Paracoccidioides brasiliensis (Pb), Penicillium citrinum (Pc), Penicillium chrysogenum (Pch), Penicillium marneffei (Pm), Talaromyces stipitatus (Ts), Trichophyton verrucosum (Tv), Uncinocarpus reesii (Ur). Sordariomycetes: Acremonium chrysogenum (Ach), Fusarium fujikuroi (Ff), Fusarium moniliformis (Fm), Neurospora crassa (Nc), Verticillium albo-atrum (Va). Basidiomycetes: Moniliophthora perniciosa (Mpe). AnLaeA, ChLae1, and AnLlmF are boxed and highlighted in yellow. B. ChLae1/LaeA amino acid alignments of ChLae1, AfLaeA and AnLaeA proteins. A. fumigatus (Accession AAR01218, Af-LaeA 349 amino acids), A. nidulans (Accession AAQ95166, AnLaeA, 374 amino acids), and C. heterostrophus (Accession JF826792, ChLae1, 292 amino acids) were aligned using ClustalW. Conserved S-adenosylmethionine binding sites are marked in red highlights, Asterisks indicate identical res [file ppat.1002542.s001.pdf]

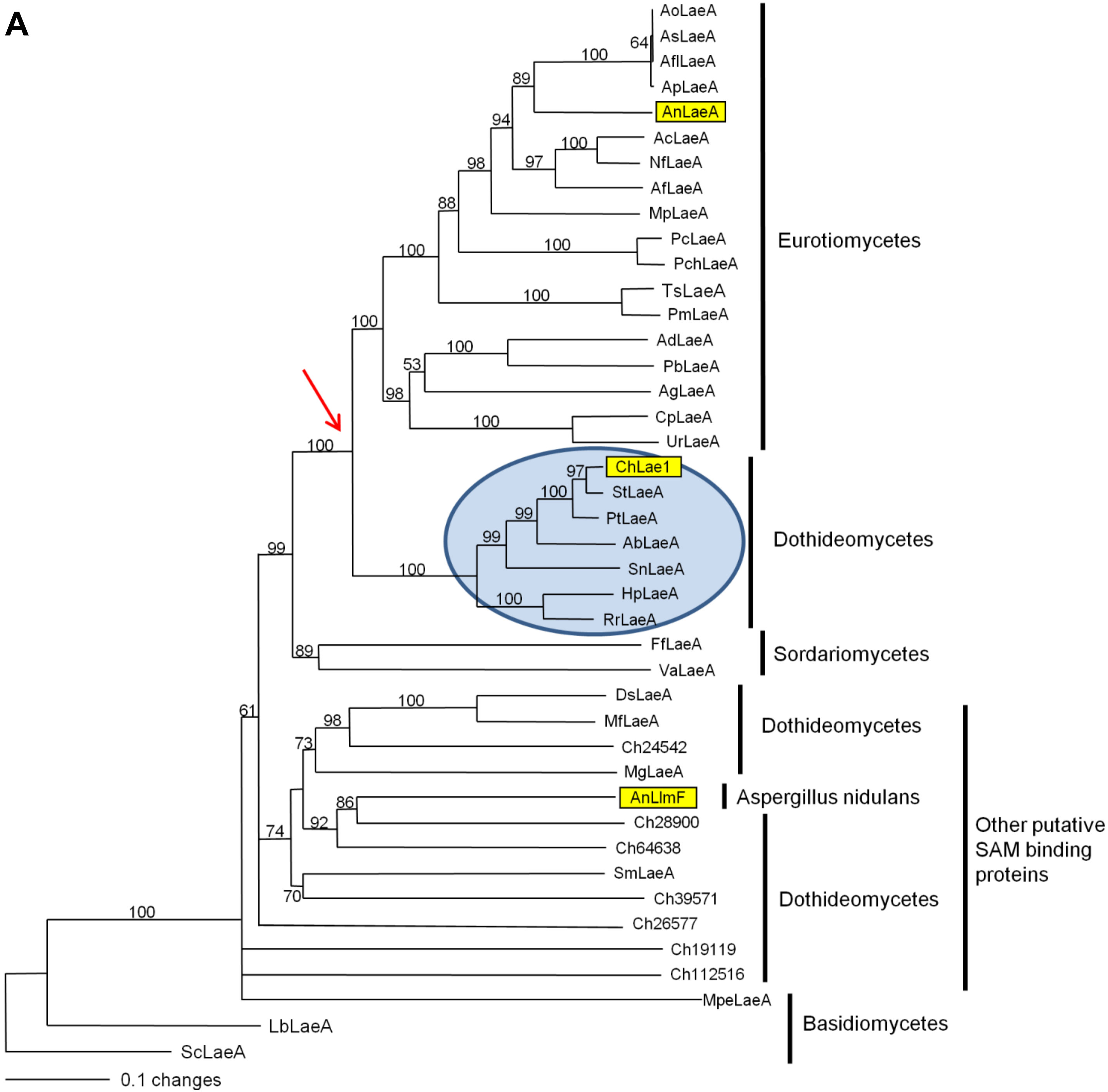
A
AoLaeA
AsLaeA
AflLaeA
ApLaeA
AnLaeA
AcLaeA
NfLaeA
AfLaeA
MpLaeA
PcLaeA
PchLaeA
TsLaeA
PmLaeA
AdLaeA
PbLaeA
AgLaeA
CpLaeA
UrLaeA
ChLae1
StLaeA
PtLaeA
AbLaeA
SnLaeA
HpLaeA
RrLaeA
FfLaeA
VaLaeA
DsLaeA
MfLaeA
Ch24542
MgLaeA
AnLlmF
Ch28900
Ch64638
SmLaeA
Ch39571
Ch26577
Ch19119
Ch112516
MpeLaeA
LbLaeA
ScLaeA
0.1 changes
Eurotiomycetes
Dothideomycetes
Sordariomycetes
Dothideomycetes
Aspergillus nidulans
Dothideomycetes
Basidiomycetes
Other putative SAM binding proteins

B

```
AfLaeA   ----------------------MLWTSIQSQTGVPLQR----ETRPLVVGTPMVPQVLIL 34
AnLaeA   MFEMGPVGTRLPAMTSPAHNHYSYHSPTSSDRGRSRQNSDAMDIQSITEREPATRYAVAG 60
ChLae1   ------------------------------------------------------------

AfLaeA   PVASISLFPSSLLFSYSCSPFFRAAVKAENGRTYHGYRKGIYMLPCDEQEQDRLDIFHKL 94
AnLaeA   GPAPWNRNGSPSMSPMYSNNSERNQFHEENGRTYHGFRRGMYFLPCDEQEQDRLDIFHKL 120
ChLae1   ---------------------------MENGRWYHGFRRGLYMYPCDEPEKDRMDIYHQF 33
                                     **** ***:*:*:*: **** *:**:**:*::

AfLaeA   FTVARVSDGLIYAPHPTN----GRFLDLGCGTGIWAIDVANKYPEAFVVGVDLAPIQPPN 150
AnLaeA   FTVARVSESLIYAPHPTN----GRFLDLGCGTGIWAIEVANKYPDAFVAGVDLAPIQPPN 176
ChLae1   FAVARRGQ-LHQAPVPSEPHLQPRILDVGCGTGIWAIDMADKYLNAEVLGLDLVNIQPEK 92
         *:*** .: *  ** *::     *:**:*********::*:** :* * *:**. *** :

AfLaeA   HPRNCDFYAPFDFESLWALGEDSWDLIHMQMGSGSVASWPNLYRRIYSHLRPG-AWFEQV 209
AnLaeA   HPKNCEFYAPFDFEAPWAMGEDSWDLIHLQMGCGSVMGWPNLYRRIFAHLRPG-AWFEQV 235
ChLae1   IPPNLRFRVPRDYESPWTLGEDSWDLIHLRMACGSVESWPELYQKIYTHLKPGTGWIEHI 152
          * *  * .* *:*: *::*********::*..*** .**:**::*::**:** .*:*::

AfLaeA   EIDFEPRCDDRSLEG-LAIRQWYQLLKQATEETMRPVAHNSRETIRNLQEAGFTEIDHQM 268
AnLaeA   EIDFEPRCDDRSLDG-TALRHWYDCLKQATAETMRPIAHSSRDTIKDLQDAGFTEIDHQI 294
ChLae1   EIDMEPRCDDYTLPPDSMLRKWYGWLADATQRAYRPIAYEHR-TRQLLQAAGFIDIQETV 211
         ***:****** :*     :*:**  * :** .: **:*:. * * : ** *** :*:. :

AfLaeA   VGLPLNPWHEDEHERRVARWYNLAISESIETMSLAPFSRVFGWPIER-IKQIAADVKSEA 327
AnLaeA   VGLPLNPWHQDEHERKVARWYNLAVSESIENLSLAPFSRVYRWPLER-IQQLAADVKSEA 353
ChLae1   IRVPYNTWPNDPHQKDIGRWYNLGLTEGLEALTFAPLTRVYHWDLNAHVRPIVEGVRREL 271
         : :* *.* :* *:: :.*****.::*.:* :::**::**: * ::  :: :. .*: *

AfLaeA   FNKEIHTYNILHIYQARKPLAN 349
AnLaeA   FNKEIHAYNILHIYQARKPLR- 374
ChLae1   CNRKIHAYNNIHIWTARRPQQ- 292
          *::**:** :**: **:*
```
