# Supplementary figures and images for: ChLae1 and ChVel1 Regulate T-toxin Production, Virulence, Oxidative Stress Response, and Development of the Maize Pathogen Cochliobolus heterostrophus
Source: PLoS Pathog. 2012 Feb 23;8(2):e1002542. doi: 10.1371/journal.ppat.1002542 (PMC3285592; doi:10.1371/journal.ppat.1002542)

A

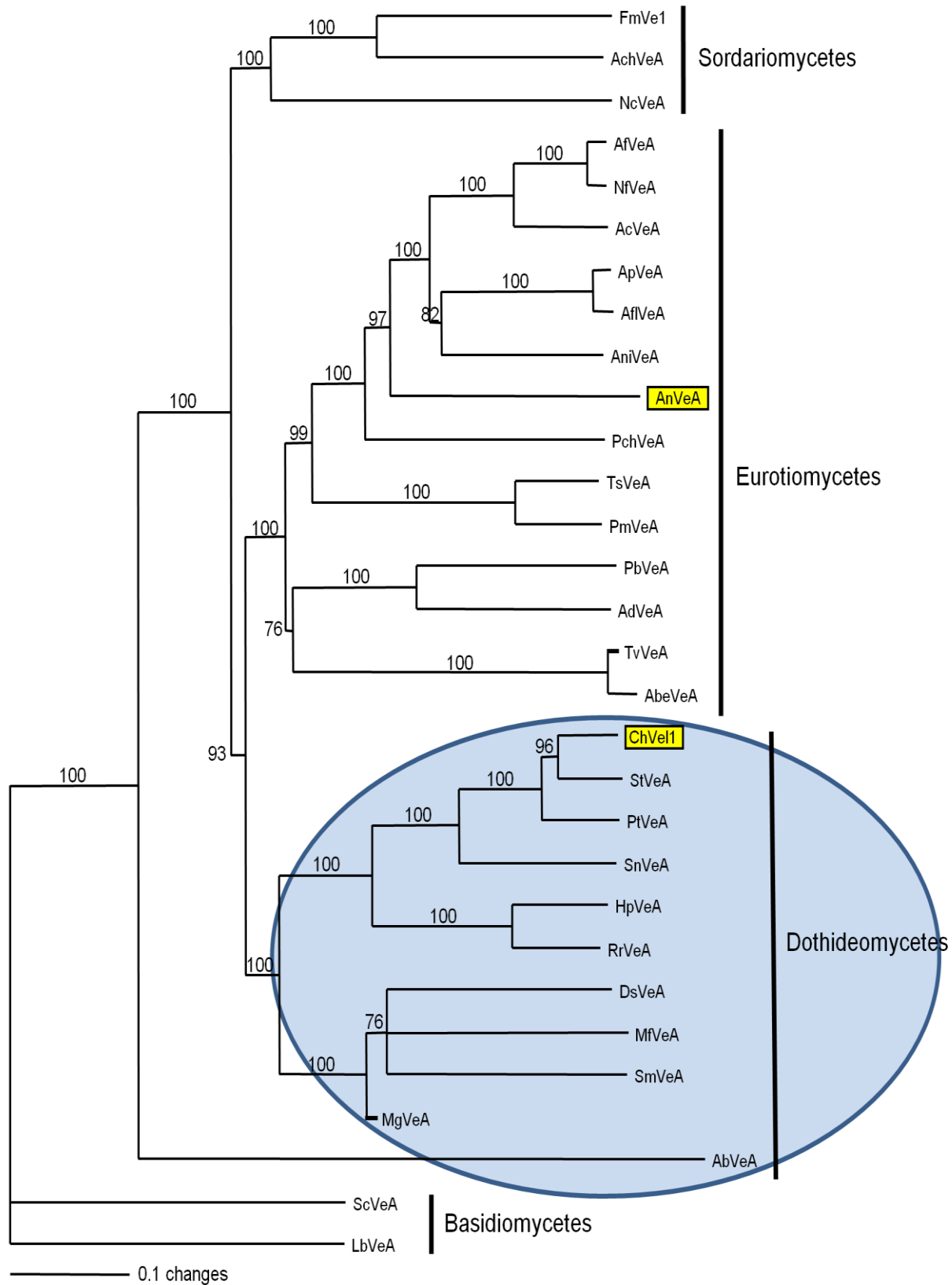

# B

[illegible]

Supplement: Figure S2 — C. heterostrophus Vel1 is an ortholog of A. nidulans VeA. A. Methods and species used as in Figure S1A. The single candidate ortholog, ChVel1, groups with single candidate VeA orthologous proteins in a well-supported group of proteins (blue oval shadow) that is sister to the Eurotiomycete group into which AnVeA falls. AnVeA and ChVel1 are boxed and highlighted in yellow. B. Amino acid alignment of ChVel1, AfVeA, and AnVeA proteins. A. fumigatus (Accession CAE47975, Af-VeA, 570 amino acids), A. nidulans (Accession AAD42946, AnVeA, 573 amino acids), and C. heterostrophus (Accession JF826791, ChVel1, 593 amino acids) were aligned using ClustalW. Putative NLS predicted by Wolf PSORT [43] is highlighted in purple. Potential α importin-dependent monopartite NLS by cNLS Mapper [44] is in blue. Potential PEST domains predicted by EMBOSS ‘epestfind’ are marked in green. Asterisks, colons, and periods as in Figure S1B. (PDF) [file ppat.1002542.s002.pdf]

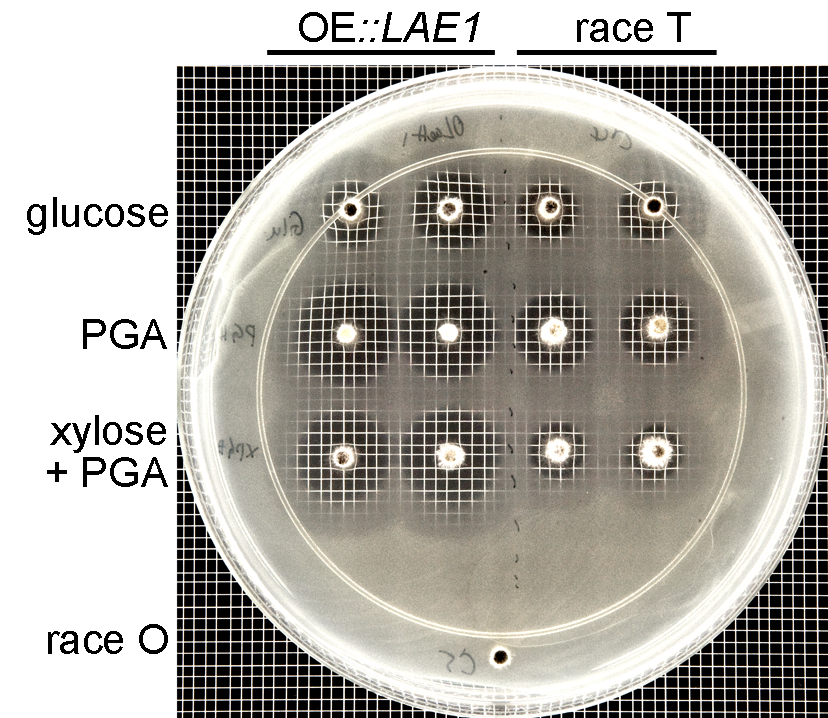

Supplement: Figure S3 — ChLAE1 overexpression strains produce more T-toxin. Plugs of each strain (OE::LAE1, WT race T strain C4) were grown on minimal medium with glucose, polygalacturonic acid (PGA) or xylose+PGA as the carbon source, in the light. Clear area (halo) indicates T-toxin production and killing of E. coli cells. Gridded paper was placed under the plates to help visualize the inner very clear area of the halo. The bottom single plug is race O, T-toxin− strain C5 control (no halo). Left two columns are two replicates of ChLAE1 overexpression strain OELaeA-1. Right two columns are replicates of race T, strain C4. In this example, the ChLAE1 overexpression strain makes more T-toxin than WT (compare two plugs from the OE strain to two from WT, second row from top), due to enhanced expression under PGA induction. Note that the ChLAE1 overexpression strain contains two copies of ChLEA1, but that, in the presence of glucose, there is less toxin than when PGA is present (compare two plugs from the OE strain, second row from top to two plugs from the OE strain, top row). (TIF) [file ppat.1002542.s003.tif]

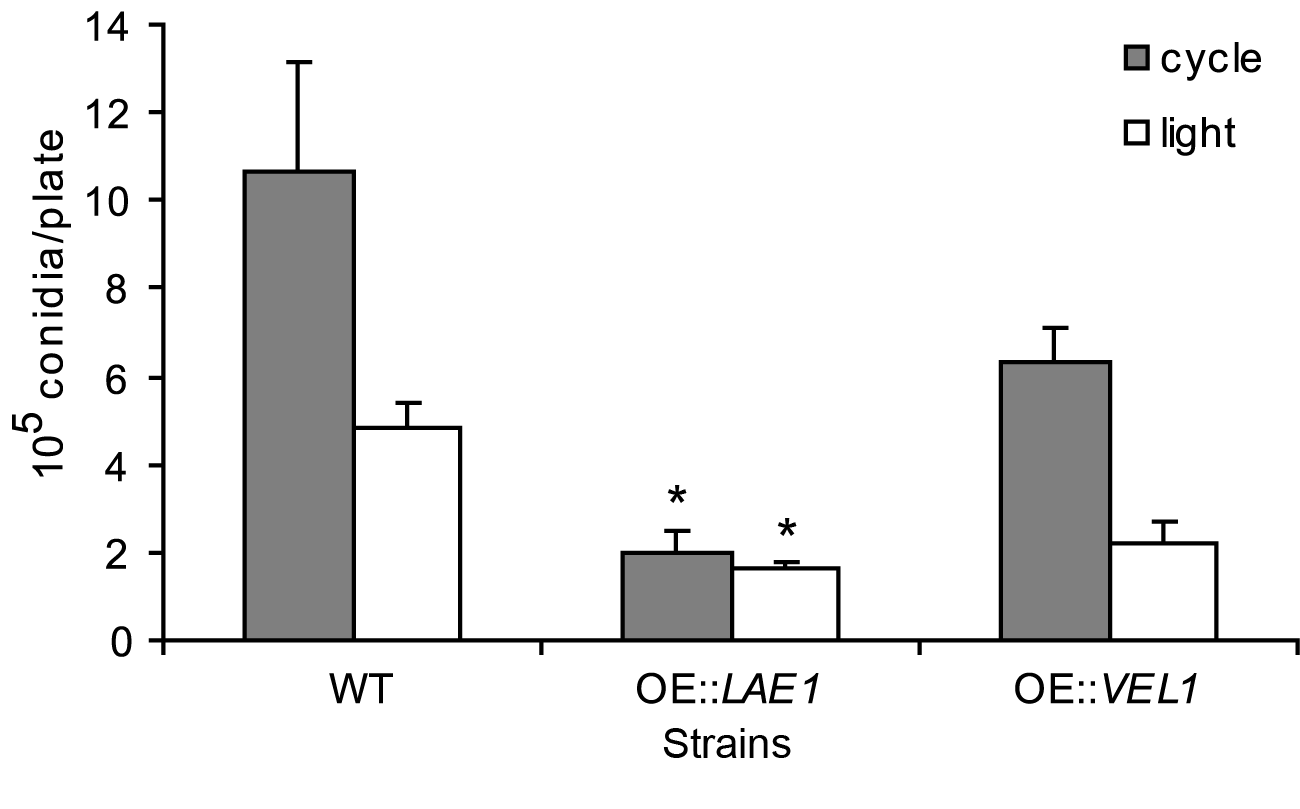

Supplement: Figure S4 — Overexpression of ChLAE1 and ChVEL1 alters asexual development. Asexual sporulation is repressed in ChLAE1 OE strains. The average number of asexual spores formed on PGA plates in constant light and in cycling conditions are shown. Error bars are standard deviation. Asterisks indicate p-value<0.05 in T-test analysis in which each strain was compared with WT C4 under the same light condition. (TIF) [file ppat.1002542.s004.tif]

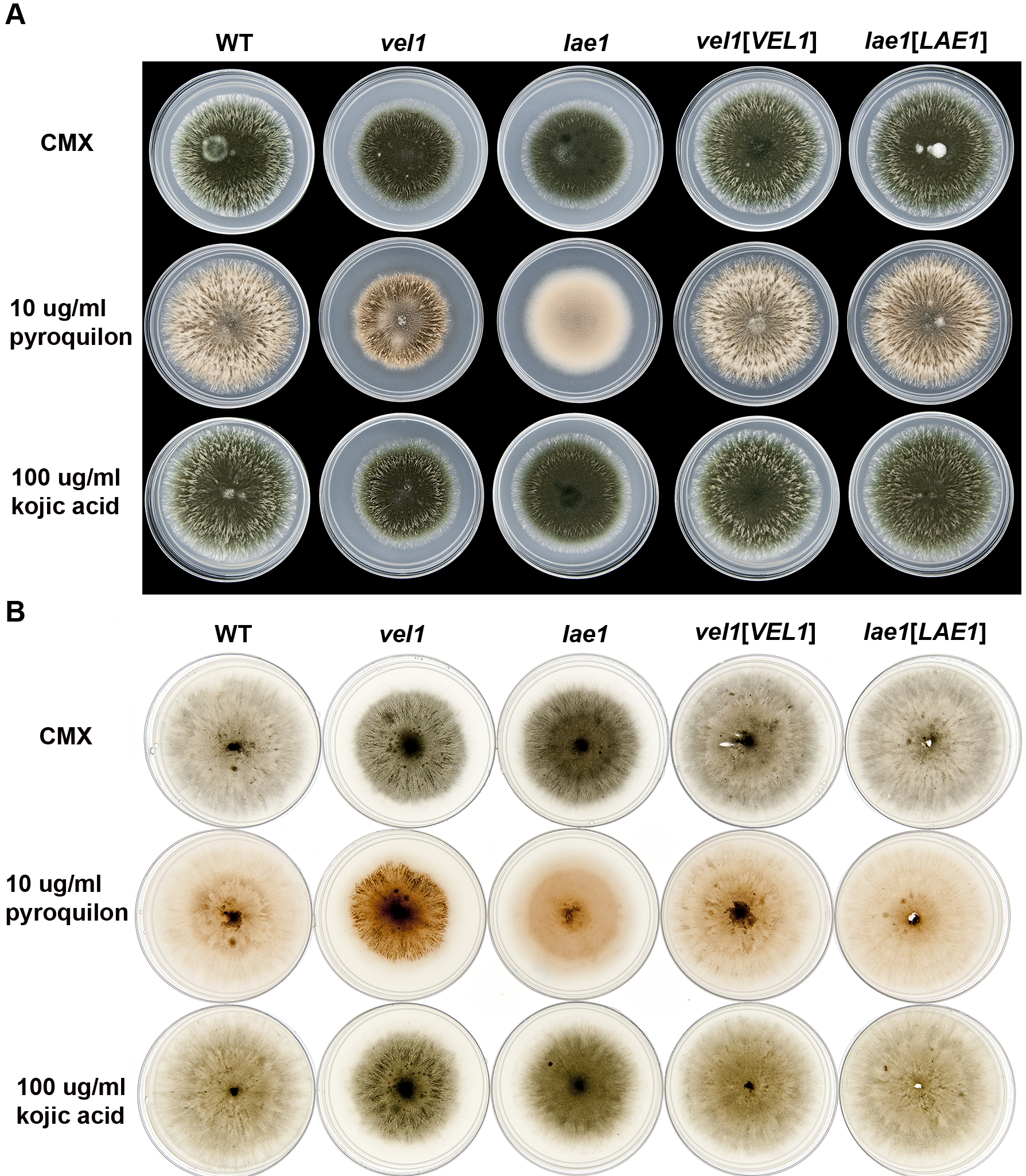

Supplement: Figure S5 — C. heterostrophus produces DHN-type melanin. A. Cultures grown on CMX and CMX containing pyroquilon or kojic acid under constant light for 7 days. Addition of pyroquilon altered the pigmentation of conidia and hyphae from dark green to light brown, while kojic acid had no effect on pigmentation. B. Culture plates from A after removal of conidia. Mycelial color was light brown for all strains tested on pyroquilon medium but unchanged on kojic acid plates. (TIF) [file ppat.1002542.s005.tif]

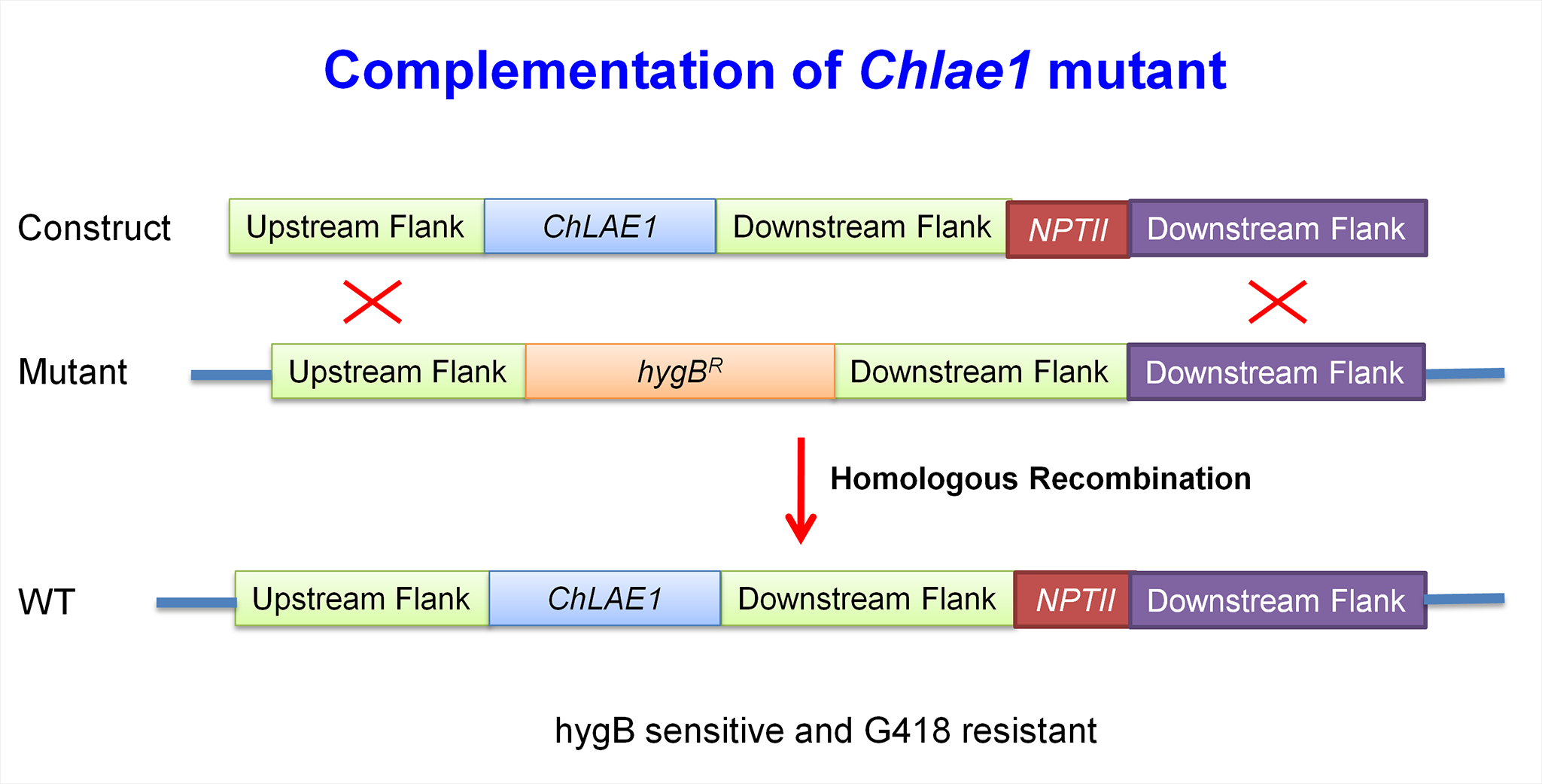

Supplement: Figure S6 — Strategy to complement the Chlae1 mutant with WT ChLAE1. The construct, described in Materials and Methods, was transformed into Chlae1 mutant ChW5. A double crossover homologous recombination event would replace the hygBR marker with the WT ChLAE1 gene and the NPTII marker (G418 resistant) at the ChLae1 locus. The resulting strains are hygromycin B sensitive and G418 resistant. (TIF) [file ppat.1002542.s006.tif]

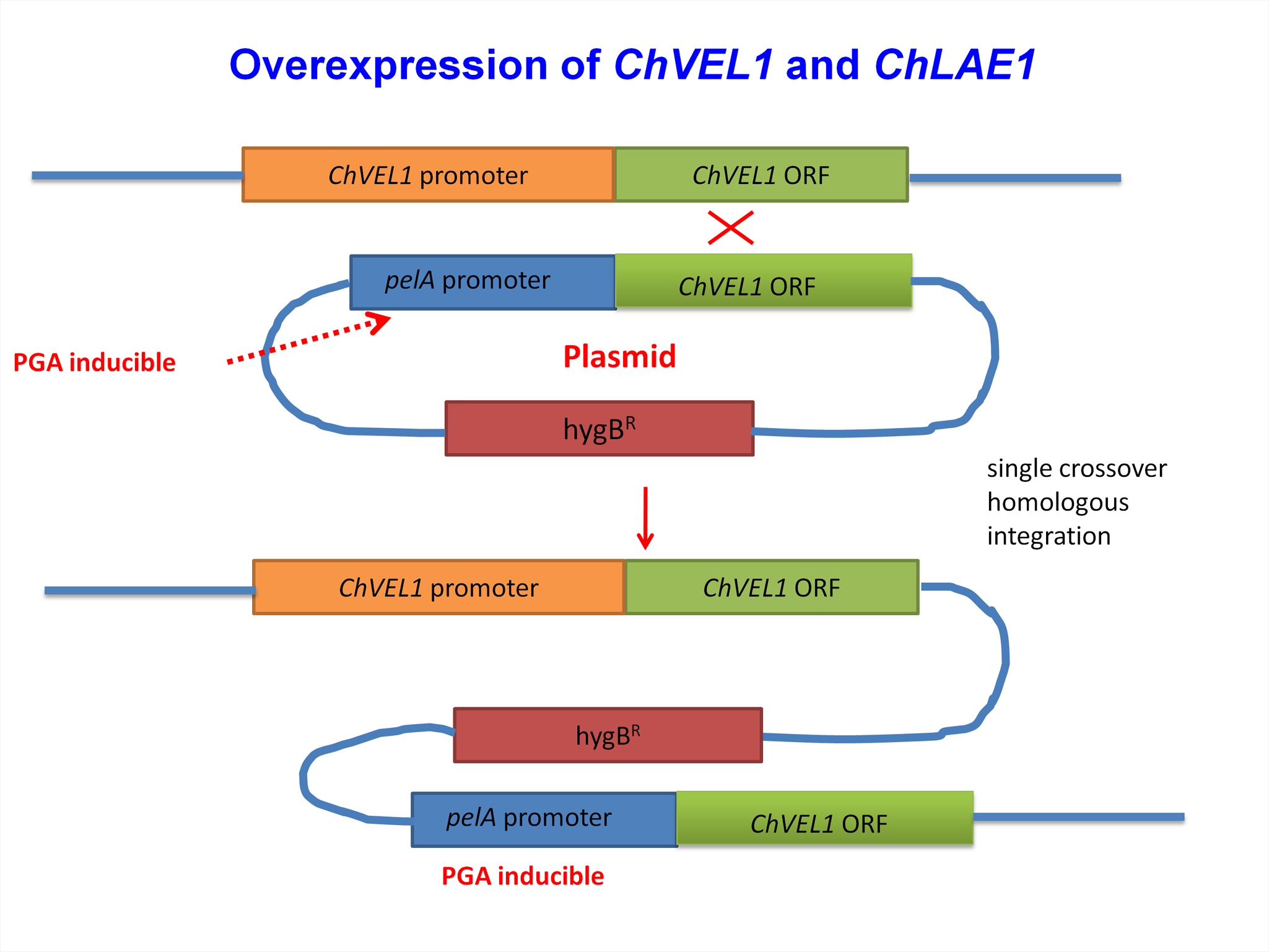

Supplement: Figure S7 — Strategy to overexpress ChLAE1 and ChVEL1. Plasmid containing either the ChLAE1 or ChVEL1 coding sequence driven by the pelA promoter (inducible by polyglacturonic acid, PGA) was transformed into WT strain C4. By a single crossover homologous integration event, a copy of PGA-inducible ChLAE1 or ChVEL1 was inserted adjacent to a copy of the same gene driven by its endogenous promoter. After induction by PGA, strains should express both the WT and introduced copies of the ChLAE1 or ChVEL1 genes. (TIF) [file ppat.1002542.s007.tif]
