# Supplementary material for: ChLae1 and ChVel1 Regulate T-toxin Production, Virulence, Oxidative Stress Response, and Development of the Maize Pathogen Cochliobolus heterostrophus
Source: PLoS Pathog. 2012 Feb 23;8(2):e1002542. doi: 10.1371/journal.ppat.1002542 (PMC3285592; doi:10.1371/journal.ppat.1002542)
Supplement: Table S1 — Primers used for this study. (DOC) [file ppat.1002542.s008.doc]

**Table S**1. Primers used for this study

| **Primer** | **Sequence 5’ to 3’** | **Description** | **PCR**  **(kb)** ^a^ | **Purpose** |
| --- | --- | --- | --- | --- |
| *VEL1*upF | CATCCCTTGCCTACCTGA | flanking region downstream of *ChVEL1* RP ^b^ | 0.78 | * *Δ *ChVEL1* ^d^ |
| *VEL1*upR | TCCTGTGTGAAATTGTTATCCGCTCCAACAAACCCAATCCAC | flanking region downstream of *ChVEL1* FP ^c^ |
| *VEL1*downF | GTCGTGACTGGGAAAACCCTGGCGTCTGCAACGAGTCAGCAC | flanking region upstream of *ChVEL1* RP | 0.72 |
| *VEL1*downR | TCTTCGGCCTATTCATGC | flanking region upstream of *ChVEL1* FP |
| NLC37 | GGATGCCTCCGCTCGAAGTA | *HygB* split marker RP | 1.2 |
| M13R | AGCGGATAACAATTTCACACAGGA | FP to amplify *HygB* |
| M13F | CGCCAGGGTTTTCCCAGTCACGAC | RP to amplify *HygB* | 1.8 |
| NLC38 | CGTTGCAAGACCTGCCTGAA | *HygB* split marker FP |
| *VEL1*upVER | GTCTTCCTTCCTTGGGCT | RP downstream of *ChVEL1,* verification primer, pair with NLC37 | 2.0 | Δ *ChVEL1* confirmation |
| *VEL1*downVER2 | TGCTTGTTGTCTCTGCCCTC | FP upstream of *ChVEL1,* verification primer, pair with NLC38 | 2.5 |
| DW101 | AGCACGCTCAACCCAATG | FP upstream of *ChVEL1,* verification primer | 1.5 | *ChVEL1* complement-ation confirmation |
| *ChVEL1*R1 | GCGGTGGGTAACAAAGTGAT | *ChVEL1* coding region, RP |
| DW26 | GTCTTCGGCTCCTTCTCATC | *ChVEL1* coding region, FP | 2.4 |
| DW102 | GCATGGGAGCAGGAGATTAC | RP downstream of *ChVEL1,* verification primer |
| *ChLAE1*FP1 | AAAGATGCAGATGGGTGGAC | flanking region upstream of *ChLAE1* FP | 0.77 | Δ *ChLAE1* |
| *ChLAE1*RP1 | CGACCCCTTTCCCAGTTTCCTCCTGTGTGAAATTGTTATCCGCT | flanking region upstream of *ChLAE1* RP |
| *ChLAE1*FP2 | CCCGTTCCATGCCAGCAAAGTCGTGACTGGGAAAACCCTGGCG | flanking region downstream of *ChLAE1* FP | 0.63 |
| *ChLAE1*RP2 | CCCACGCTAGAAACCTTCAG | flanking region downstream *ChLAE1* RP |
| *ChLAE1*U1 | GGATGGGTCCCAGAGGTG | upstream of *ChLAE1* verification primer, FP, pair with NLC37 | 2.0 | Δ *ChLAE1* confirmation |
| *ChLAE1*D1 | ATACTGCCTGATCCGTGTCC | downstream of *ChLAE1* verification primer, FP, pair with NLC38 | 2.7 |
|  |  |  |  |  |
| DW270 | AAGATCACTGGAACAACTGGCATGCCCACGCTAGAAACCTTCAG | RP to amplify *ChLAE1* sequence with PtrpC tail for complementation, pair with *ChLAE1*FP1 | 2.7 | *ChLAE1* complement-ation |
| DW69 | CATGCCAGTTGTTCCAGTG | FP TrpC promoter | 2.7 |
| DW70 | ACCTCTAAACAAGTGTACCTG | RP TrpC terminator |
| DW271 | GCACAGGTACACTTGTTTAGAGGTGGCAAAGTGGCTCGGCAT | FP to amplify *ChLAE1* downstream flanking sequence with TtrpC tail for complementation | 0.7 |
| DW272 | GCCAAAACAACTTCACCAC | RP to amplify *ChLAE1* downstream flanking sequence for complementation |
| TtrpCend | GTGAATGCTCCGTAACACCCAATAC | FP pUCATPH, Terminator of TrpC | 1.2 | *ChLAE1* complement-ation confirmation |
| DW273 | TGTCTGTGTCTGTTCCTTCG | RP for confirmation *ChLAE1* complementation |
| *ChLAE1*F1 | GGCCGCTGGTATCATGGCTTCCG | 11 bp downstream of start codon of *ChLAE1* | 1.2 |
| *ChLAE1*R1 | AGCATCGGCCCAGTAACATCAAAC | near stop codon of *ChLAE1* |
| DW28 | TGCAggatccATGTCCAACATTGTTGTAAG | VeA OE FP at ATG with *Bam*HI cut site | 2.8 | *ChVEL1* OE ^e^  plasmid construction |
| DW29 | TCGGaagcttGTTTCACCTTTGGGGTAAATC | VeA OE RP at +287 bp with *Hin*dIII cut site |
| DW30 | CGAGggatccATGGAAAATGGCCGCTGG | LaeA OE FP at ATG with *Bam*HI cut site | 2.0 | *ChLAE1* OE plasmid construction |
| DW31 | CGTCaagcttCTATACCGTCATGCCGAG | LaeA OE RP at +376bp with *Hin*dIII cut site |
| DW38 | AGATGGTCAACGCTGCTTAC | pelA promoter primer for confirmation of OE strains, 141 bp away from *Puv*II site | 2.9 | *ChVEL1* OE confirmation |
| DW39 | CGCAGTTTTGGGATTTGAG | 94 bp away from DW29 for confirmation of *ChVEL1* OE strains with DW38 |
| DW40 | ACTCTAGAACATCCTCGACG | 215 bp away from DW31 for confirmation of *ChLAE1* OE strains with DW38 | 2.2 | *ChLAE1* OE confirmation |
| DW41 | GCTGGTTGGAGAAGTATGAG | Trehalose RP for confirmation of Promoter 1 integration | 1.0 | *ChVEL1* and *ChLAE1* OE confirmation |
| DW42 | ACGAACCCTGAATGACAAG | PelA promoter RP for confirmation of Promoter 1 integration, pair with DW41 |

^a^ PCR (kb) = PCR product length in kb; ^b^ FP = forward primer; ^c^ RP = reverse primer ^d^; Δ = gene deletion;

^e^ OE = overexpression
